# PyMolGen: Database-Driven Molecular Generation of Drug-Like Compounds

**DOI:** 10.1021/acs.jcim.6c00689

**Published:** 2026-06-16

**Authors:** Bruno N. Falcone, Michael J. Hutcheon, Jaffer M. Zaidi, Michael J. Stocks, Kirsty E. Hamill, Craig Jamieson, Huw J. Williams, David S. Palmer, Andrew Baxter, Stephen D. Pickett, Jonathan D. Hirst

**Affiliations:** † School of Chemistry, 6123 University of Nottingham, University Park, Nottingham NG7 2RD, United Kingdom; ‡ Biodiscovery Institute, School of Pharmacy, University of Nottingham, University Park, Nottingham NG7 2RD, United Kingdom; § Department of Pure and Applied Chemistry, 3527 University of Strathclyde, Glasgow G1 1XL, United Kingdom; ∥ Medicines Research Centre, 1929 GlaxoSmithKline, Stevenage SG1 2NY, United Kingdom; ⊥ Department of Cheminformatics, Research Technologies, GlaxoSmithKline, Stevenage SG1 2NY, United Kingdom

## Abstract

We present a rule-based approach for the generation of new molecules, which derives combination rules between molecular fragments from a user-provided database, and is useful for constrained optimization at defined vectors. The molecules generated are statistically similar to the database provided. Compared to existing machine-learning approaches this affords the user a greater level of control of the molecules generated, which is a common desideratum in drug discovery programs. This is complemented by the use of a build probability score for each generated molecule, which corresponds to the likelihood of its existence in a hypothetical exhaustive enumeration of the source database fragments. The method is applied to the generation of derivatives of a 3,5-dimethyl-4-phenylisoxazole parent structure, a core of current interest for the development of inhibitors of bromodomain-containing protein 4.

## Graphical abstract

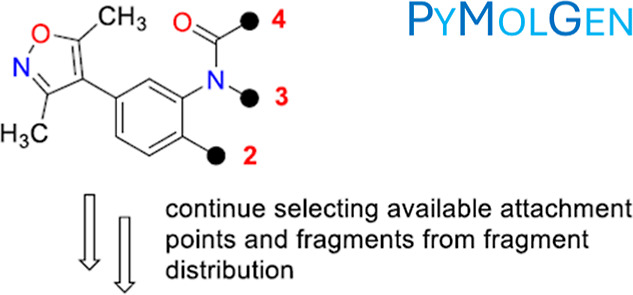

## Introduction

The exploration of chemical space for drug discovery has been recognized as a daunting task; it is estimated that there are up to 10^60^ synthetically accessible drug-like compounds, of which only 10^8^ have been synthesized thus far.^1^ As traditional *in vivo* and *in vitro* libraries can only cover a very small fraction of this space, efficient computational methods for molecular generation are therefore necessary to sample novel and relevant compounds.

There have been two main approaches to molecular generation: rule-based and machine learning-based. Some rule-based methods^2^ focus on systematic database generation. GDB^3,4^ and GDB-13,^5^ for example, compile the databases through enumeration over connected graphs using a limited set of atoms (C, N, O and F in GDB and C, N, O, F, S and Cl in GDB-13) for comprehensive coverage of relevant molecules under a certain size, imposing molecular structure constraints to avoid undesirable molecules. Although these databases have been employed in virtual high-throughput screening,^2^ these atom-based methods tend to produce molecules of high synthetic complexity. To avoid this issue, fragment-based approaches have often been favored. Mauser and Stahl used fragments generated from retrosynthetic rules from drug-like molecules,^6^ which were filtered and combined using a set of compatibility rules. The BRICS method^7^ utilized the set of fragmentation rules devised in RECAP,^8^ which are based on simple and widely used reactions such as amide and aryl couplings.

In terms of methods based on machine learning, deep learning has emerged as a powerful tool in molecular design. Gómez-Bombarelli et al.^9^ used a method to convert discrete representations of molecules to and from a multidimensional continuous representation in order to search for optimized drug-like molecules. Jin et al.^10^ introduced an autoregressive coarse-to-fine decoder to add structural motifs, extracted from frequently occurring substructures, one at a time. This approach is particularly suited for polymeric molecules and is claimed to outperform traditional atom-by-atom or substructure-based methods. To address the lack of chemical diversity observed with some of these models, Blaschke et al.^11^ developed a reinforcement learning approach that uses recurrent neural networks (RNNs) for molecule generation and incorporates a “memory unit”, using similarity measures, such as the Tanimoto index, to explore diverse areas of chemical space. In addition, Zhavoronkov et al.^12^ developed a model, generative tensorial reinforcement learning (GENTRL), which combines reinforcement learning, variational inference and tensor decompositions into a generative two-step algorithm. GENTRL optimizes for synthetic feasibility, novelty, and biological activity and it was applied to the discovery of potent inhibitors of discoidin domain receptor 1 (DDR1) and the new compounds were successful in biochemical, cell-based and mice models. Methods for the construction of fragment libraries in different chemical environments include an approach^13^ based on matched molecular pair analysis, and an algorithm that determines interchangeable fragments from the ChEMBL database database by storing the context radius (typically of length 3) between fragments. This information is used to effect “chemically reasonably mutations” (CReM) through Mutate, Grow and Link operations.^14^ There also various studies that use such fragment spaces to enumerate a chemical space iteratively or optimize a scoring function, for example using a genetic algorithm.^15^ Loeffler et al.^16^ have introduced REINVENT 4, a molecular generation method that uses recurrent neural networks and transformers architectures. The authors provide an overview of the various AI-driven models for generative molecular design, including variational autoencoders (VAEs), generative adversarial networks (GANs), RNNs, transformers, and diffusion models. All these methods have their relative merits and there is no one solution that uniformly outperforms the others.

There has been recent debate about the ability of AI algorithms to generate “novel” molecules.^17^ However, in most applications in a drug discovery context the starting point is a hit from a screening campaign or a knowledge-based approach. Thus, the problem is one of local optimization addressing the challenge of optimizing the series according to the requirements of the program (oral/inhaled, CNS/non-CNS, etc.). Therefore, a key metric for a molecular generation scheme is the confidence with which its output can be fed into a screening campaign—hence, the ability to closely control this output is desired.

In this work, we have devised a rule-based approach for the generation of new molecules, which derives the combination rules between fragments from a user-provided database, and is particularly useful for constrained optimization at defined vectors. The advantage over existing machine-learning approaches is that the molecules generated are statistically similar to the database provided, affording the user a greater level of control of the molecules generated. This is enhanced by the use of a build probability score for each generated molecule—reflecting the likelihood of its existence in a hypothetical exhaustive enumeration of the source database fragments. The conditioning of fragment selection on the underlying probability distribution can be employed either to bias generation output toward more target- or medicinally relevant structures, or actively to avoid inappropriate levels of structural novelty in generated output. The latter case allows for a systematic and efficient enumeration of chemical spaces that would be computationally prohibitive to exhaustively enumerate and filter.

We have applied the method to the generation of derivatives of the 3,5-dimethyl-4-phenylisoxazole parent structure (Figure 1), which is a promising core in the development of inhibitors of bromodomain-containing protein 4 (BRD4).^18,19^ Substitution was explored at the *para* and *meta* positions of the benzene ring.

**Figure 1:**
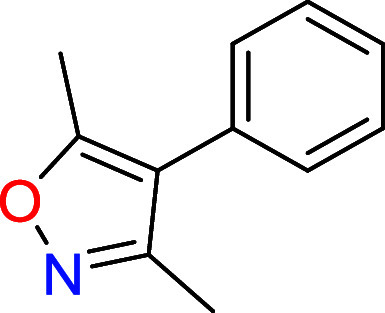
3,5-dimethyl-4-phenylisoxazole (for simplicity, referred to as phenylisoxazole in the text).

Bromodomains are important regulators of the epigenome through their ability to selectively recognize and bind to ε-*N*-acetylated lysine (KAc) residues on the terminus of histone tails. This binding interaction is a pivotal mechanism in numerous cellular processes and is associated in many downstream signaling pathways involved in a diverse range of inflammatory diseases, hematological malignancies and solid tumors. The discovery of BRD4 inhibitors, with various chemomimetics of the *N*-acetylated lysine residues, such as dimethyltriazoles, dimethylisoxazoles, acetamides and pyridines, thus, continues to be an active area of research.^20–24^

## Methods

Our protocol is written in python and is called PyMolGen. The goal is to generate drug-like molecules that are synthetically tractable and which satisfy the ‘rules’ of organic chemistry, but without needing to hard-code these rules. The protocol is part of a larger design-make-analyze-test (DMTA) cycle, involving a cheminformatics-led compound selection, whereby proposed new compounds generated by PyMolGen are scored using ML models to predict activity and physiochemical properties, with promising compounds taken forward for synthesis. Figure 2 shows a schematic of the wider DMTA workflow.

**Figure 2:**
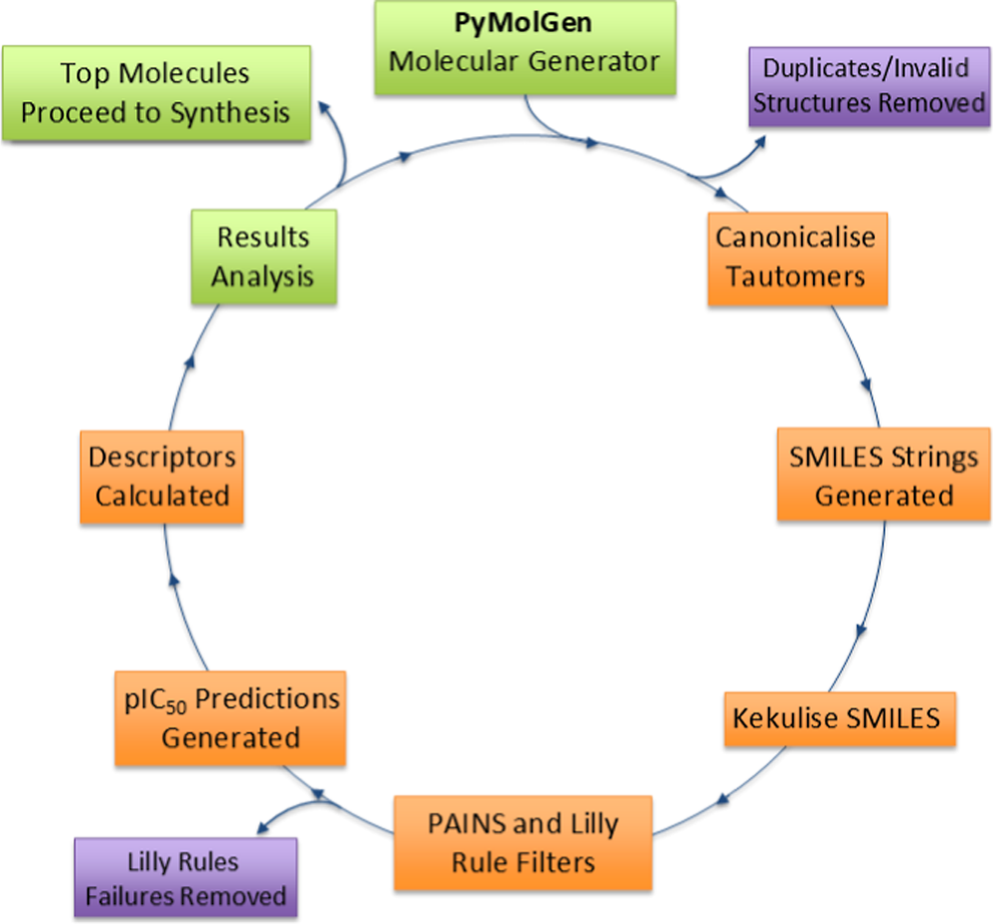
DMTA cycle with PyMolGen as the molecular generator. Components involving human input are shown in green, those that are fully automated are in orange, and steps where compounds are removed are shown in purple.

The molecular generation algorithm in PyMolGen decomposes the compounds from a source database into individual fragments and derives the combination rules between these fragments from the fragment disconnection distribution. The code allows for flexibility in the fragmentation rules employed, but we chose to break single bonds that are not part of cycles. Given that the carbonyl group is ubiquitous in drug-like compounds, we chose to extend the fragment definition around carbonyl groups by not allowing the fragmentation between the carbon atom in a carbonyl and other heteroatoms bonded to it (Figure 3a). For example, ester or amide bonds are not fragmented, but ketones are fragmented into a carbonyl and the alkyl or aryl groups bonded to the carbonyl. Following this fragmentation, the bonding pattern between fragments is stored as a map from vectors (***i***, ***j***, ***k***, ***l***) to the frequencies of each bonding combination (Figure 3b). The indices (***i***, ***j***, ***k***, ***l***) represent a bond between fragment ***i*** and fragment ***j*** through atom ***k*** in ***i*** and atom ***l*** in ***j***. The frequencies of those bonds in the database are stored and used throughout the code as proxies for both medicinal chemistry relevance, and for synthetic accessibility.

**Figure 3:**
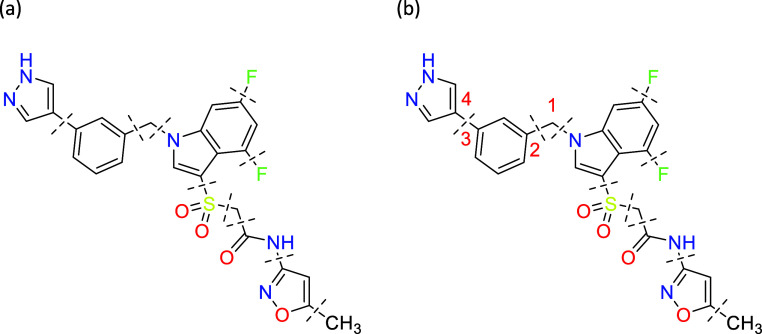
(a) Fragmentation of an exemplar compound from the ChEMBL database. Only exocyclic single bonds are broken. Heteroatoms adjacent to carbonyls are retained as a composite fragment, as in the amide in this compound. (b) Bonding information stored between fragments: atoms 1 and 3 are considered equivalent due to symmetry of the 1,3-disubstituted benzene. The full bond index reflects the connectivity of these atoms to carbon atoms within the pyrazole and methylene fragments, respectively.

### Molecular Representation

Molecules are described as a NetworkX graph with atoms as nodes and bonds as edges. Atoms at which a molecule can bond with another are labeled as attachment points. The free valence points is a list of atoms in a fragment that can bond to further fragments and if an atom can bond to *n* fragments then that position is listed *n* times. The *valence* is the total bond order attached to an atom, e.g., 3 for a carbonyl carbon. The *hybridization* is the number of neighbors plus the free valence of that atom, e.g. Four for the sp^3^ carbon in CH_2_ (with two free valence points) and 3 for the sp^2^ carbon in C=O (with two free valence points). This molecular representation allows for an efficient and accurate tracking of the molecular fragmentation and building process.

### Fragmentation of the ChEMBL Database

The 2.3 million compounds from the ChEMBL database^25^ were converted to SDF format using open babel^26^ and were fragmented by breaking single bonds which are not part of cyclic ring systems using the fragment_mol.py code in PyMolGen. To increase the throughput of the computation, this fragmentation was parallelized for batches of 10,000 ChEMBL compounds and the fragment information was aggregated using fragment_mol_combine.py, while removing infrequent fragments that had a frequency of less than five in each batch.

### Pruning of Fragment Database

The fragment database was pruned by removing complex and reactive structures (Figure 4), using fragment_mol_combine.py. Fragments containing atoms other than Cl, Br, I, O, C, F, N, S or H were removed. For fragments containing three aliphatic rings (as defined with the CalcNumAliphaticRings function in RDKit^27^), small cages were kept (structures with less than 12 heavy atoms), while the rest of the structures were removed. Structures with more than three aliphatic rings were all removed. Disulfides (defined in the algorithm as sulfur bonded to sulfur), sulfones (sulfur bonded to two oxygen atoms) and all sulfur atoms which were not in cycles were also removed. The coarse filtering of complex fragments was commensurate with the intent of PyMolGen to generate structures that would be accessible with a palatable amount of chemical resource for exploratory discovery compounds. Complex composite ring systems and macrocycles are more likely to require lengthy bespoke synthesis, particularly when exocyclic modifications are made. This approach could be further refined through cross-referencing of the unfiltered fragment database against reagent catalogs.

**Figure 4:**
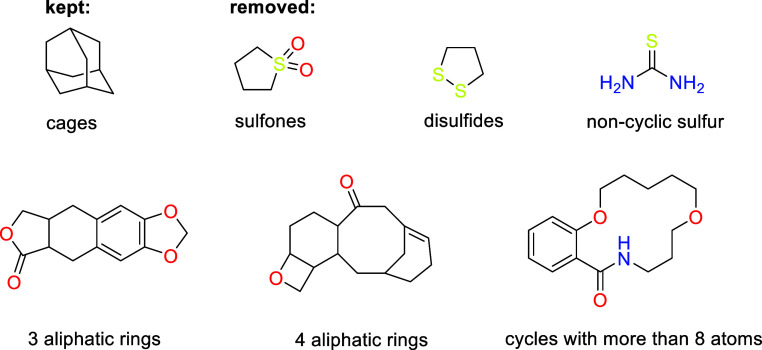
Fragments that were analyzed due to complexity, toxicity or reactivity. Only cages with less than 12 heavy atoms were kept, all other fragments shown were removed.

Three model generations using PyMolGen were carried out, all employing the parent phenylisoxazole as the starting point, with substitutions allowed at the *meta* and *para* positions.

### Preparation of the Parent Structure

Once the fragment database is compiled, it is possible to start the construction of new molecules following the derived combination rules. First, a parent compound must be defined. This would usually be a core structure that was identified in a medicinal chemistry program, i.e., a substructural motif which is considered key for the activity against a target, and which is to be derivatized as part of a hit discovery or lead optimization program. In this example, 3,5-dimethyl-4-phenyl-isoxazole (for simplicity, later referred to as phenylisoxazole in the text) was chosen as the parent compound. However, the parent structure may not necessarily be present in the original database as an independent fragment (this is the case for phenylisoxazole, as it consists of four fragments: isoxazole, benzene and two methyl groups). In such a case, the attachment points (i.e., free valence points from which further elaboration is desired) are selected for the parent structure. These can be guided by project context, such as consideration of relevant molecular modeling, or stereoelectronic or synthetic considerations of the core structure. If the parent structure is not present in the fragment database, the attachment points must be defined as equivalent to a fragment present in the database (using chemical intuition) in order to set the attachment probabilities.

### Random Generation of 138 Million Molecules

In this example, a benzene ring with one available attachment point was chosen as the equivalent fragment for each of the substitution points of the phenylisoxazole parent structure. The code then chooses one of the substitution points at random, searches for the allowed bonds to the reference fragment in the database and picks one of them at based on a probability given by the bond frequencies as originally derived from the database. In the example shown in Figure 5, an amide group was chosen for attachment to the *meta* position of the core structure, with a direct bond between the benzene ring and the amide nitrogen.

**Figure 5:**
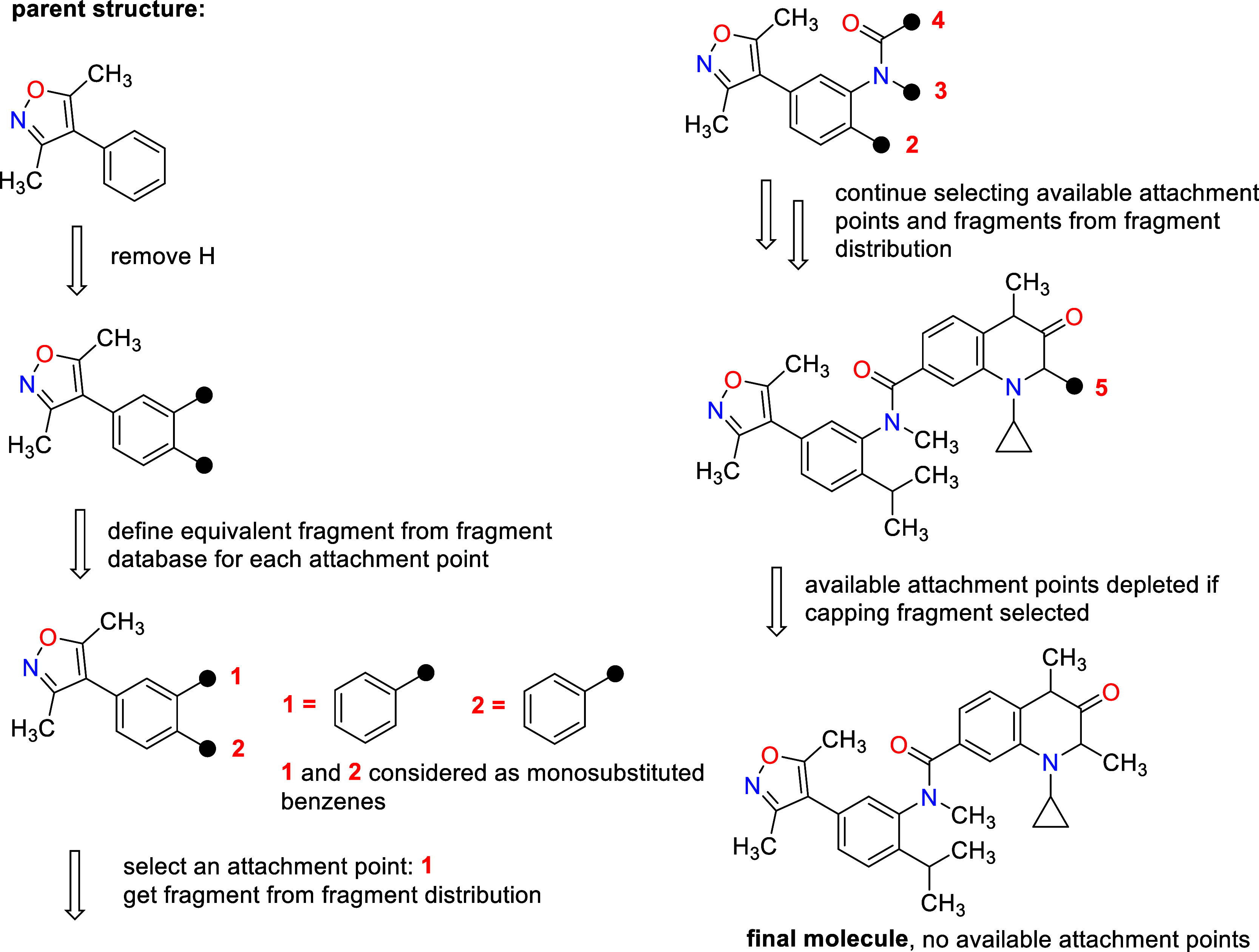
Example of a molecular generation from parent structure phenylisoxazole with functionalization at the *meta* and *para* positions.

The resulting structure has two available attachment points: the remaining attachment point from the parent structure at the *para* position of the benzene ring, and a new attachment point at the carbonyl of the newly added amide group. From these attachment points, one is chosen at random, the bond frequencies are evaluated, and a bond is formed to a fragment sampled from the eligible subset of the database, weighted by the underlying bond frequencies. This process continues until the molecule does not have any more available attachment points (*i.e*., by adding capping fragments which do not have further attachment points) or it can additionally be stopped based on user-specified intervention criteria, *e.g*., a maximum allowed molecular weight.

Compounds were generated using the random generator (fragment_builder.py, which uses the Molecule class, based on NetworkX,^28^ to describe molecules as networks of atoms) by running 100 independent processes in parallel for 72 h. The InChI codes of all molecules were generated and duplicates removed.

Several configuration options can be adjusted for the generation of new molecules. A generation run can be controlled by the number of molecules required or by processing time, and intermediate molecules can be saved after each incremental fragment addition with H-capping of any open attachment points. This also makes it possible to track the way that each final molecule was constructed by the algorithm. By default, the code may produce repeats of molecules, but an option can be selected to only save distinct structures. Other options are available (see the code documentation for full details). Initial runs were performed with the *intermediates* option to keep a record of intermediate molecules as new fragments were added, and with the *cap* option, to retain the final molecule where the last fragment addition increases the overall molecular weight above the specified budget of 500 Da.

### Fragment Graph Description of Molecules

To speed up the generation of molecules, molecules were described as a graph of fragments, by using the FragmentGraph and FragmentGraphNode objects (Figure 6). Each fragment is represented as a FragmentGraphNode, with a certain number of attachments points and bonds with other FragmentGraphNodes (Figure 6). A mapping between the FragmentGraph objects and the Molecule objects is required to convert between the two representations, for molecular fragmentation and building purposes. The free valence points property keeps track of available attachment points to make further bonds with other fragments. This approach is about 50 times faster for molecule generation.

**Figure 6:**
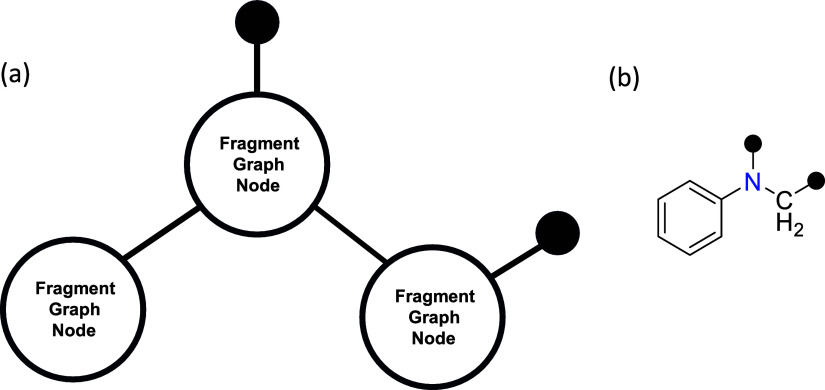
(a) Structure of a molecule within PyMolGen represented as a FragmentGraph connecting different FragmentGraphNodes with bonds between them, and free valence points where further FragmentGraphNodes can be added. (b) Molecule corresponding to that FragmentGraph, with free valence points shown as black circles.

### Systematic Generation with the Fragment Graph Approach

The chemical space accessible to PyMolGen depends on the fragment database and nature of the initial core attachment points. In most cases, this is vast, and we devised a method to approximate the “realistic” output of an exhaustive PyMolGen enumeration, excluding the fragment combinations with a vanishingly small probability of being sampled from the underlying distributions. To this aim, we introduced the concept of a build probability, which indicates the likelihood of generating a specific structure. It has been designed as follows: for the addition of a new fragment A to a parent structure X,

*X* + *A* → *X*–*A* the probability of generating that specific structure becomes

$$P\left(XA\right)=\frac{P\left(X\right)P\left(X-A\right)}{N_{free.valence.points}}$$

where *P*(*X*) is the probability of *X*, *P*(*X*–*A*) is the probability of making the *X-A* bond (as derived from the original database) and *N*_free.valence.points_ is the number of available attachment points, i.e. the total free valence of *X*.

For the addition of a single fragment to a parent structure (referred as a “+1” addition), the build probability *P* corresponds exactly to the probability of generating a specific +1 fragment structure, i.e., if all possible +1 molecules were enumerated from that parent structure, the distribution of each structure would correspond to the build probabilities.

Upon addition of further fragments, the build probability of the final compound corresponds to the product of the build probabilities of each step, so that

$$P\left(XF\right)=\prod_{i}P\left(X-i\right)$$

where *P*(*XF*) is the probability to make the final product *XF*, and *P*(*X*–*i*) is the probability of adding a further fragment *i* over the previous structure. In this way, the generation can be understood as a Markov process, with events corresponding to fragment addition.^29^

The systematic molecular generation is achieved through two functions: a *recursive* function (extend_molecule_recursive) and a *depth* function (extend_molecule_list_depth). The recursive variant is used to generate molecules up to a specified build_probability threshold. The function works by iterating over all available free valence points in a molecule, then iterating over all possible bonds (*i.e*., possible further fragments that can be added according to the construction rules), calculating the build_probability of the resulting molecule and if that probability is above the specified threshold, then the fragment is added and the function is called again in a recursive fashion. Alternatively, the *depth* variant allows for addition of a specified number of fragments to the parent structure, *e.g*., if depth two is used, a maximum of two fragments will be added. This function receives a list of molecules (comprising only the parent structure when initiating the first fragment addition) and adds one fragment to each molecule in the list by calling extend_molecule_list. This creates a new list of molecules with one further fragment added, and this process will be repeated until the desired number of fragments (depth) has been achieved. Fragments are added in order of decreasing bond probabilities up to the build probability threshold.

An exhaustive generation of molecules with a threshold of 10^–10^ for the build probability was carried out using the *depth* option in fragment_molecule_builder.py. To reduce RAM usage and speed up the generation, first all depth 1 molecules within the build probability threshold were generated, and this set of molecules was split into 1000 independent sets with similar sums of build probabilities by using the split_molecule_list.py code in PyMolGen. Recursive generation was continued on these independent sets in parallel, and the resulting outputs combined based on depth with duplicate molecules removed by their InChI codes.

This implementation avoids the arbitrary decision in the initial PyMolGen variant on when to terminate the generation run, knowing that as run time increases, the output will increasingly be duplicate rather than novel structures, with increasingly redundant use of compute to generate and discard these duplicates.

### Random Generation of 2.1 Billion Molecules with the Fragment Graph Approach

2.1 billion distinct compounds were generated, with the second version of the generator using the *random* option (fragment_molecule_builder.py, which uses the FragmentMolecule class to describe molecules as graphs of fragments) by running 100 independent processes in parallel for 24 h. The initiating parent fragment was phenylisoxazole, with the initial attachment points set to mimic a benzene fragment with a single attachment point in the fragment database. The build probabilities, molecular weights and number of added fragments (depths) were saved for all molecules using the *str* representation of the FragmentMolecule class. To avoid generation of a large number of duplicates, the minimum depth to store was set to 3. The maximum depth was 12. (A generation of depth 1 and 2 can be performed by full enumeration and it is estimated that a maximum of 70 million compounds of depths 1 and 2 could be generated for this parent and fragment pool). Unique sets for each depth were obtained from deduplication of InChI codes.

### QSAR Model

A regression QSAR model was built based on experimental data from ChEMBL for BRD4 inhibitors. The model to predict pIC_50_ is 2D ligand based and uses RDKit descriptors for molecular representation. It was developed using a DeepChem Graph Convolutional Neural Network^30^ (GCNN) trained on ca. 2800 molecules and associated assay data. Further details will be published elsewhere.

### Filters

The generated molecules were filtered according to extended Lipinski rules^31^ using calculator.py: maximum Molecular Weight (MolWt, RDKit) of 500 Da, a maximum of two chiral atoms (OEIsChiralAtom function from OpenEye), maximum polar surface area (OEGet2dPSA, OpenEye) of 140 Å^2^, a maximum of seven rotatable bonds (OEGetRotatableBondCount, OpenEye), maximum number of H-bond acceptors (IsLipinskiAcceptor, OpenEye) of ten, maximum number of hydrogen-bond donors (IsLipinskiDonor, OpenEye) of five, a log *P* (OEGetXLogP, OpenEye) between 0.5 and 5, and a maximum Property Forecast Index^32^ (PFI) of eight, where the PFI is the sum of the log *P* and the number of aromatic rings (CalcNumAromaticRings, RDKit); log *P* is used instead of log *D* as in the original PFI. These coarse filters were selected to generate output structures more likely to have desirable physicochemical and pharmacokinetic properties for an orally administered small molecule, and to reduce outputs requiring highly convoluted synthesis.

### Rules

The generated molecules were filtered according to the Eli Lilly MedChem rules^33^ using rules.py. These rules consist of a set of criteria that aim to avoid issues in the development of drugs, such as reactivity (e.g., acyl halides), interference with assay measurements (fluorescence, absorbance, quenching), activities that damage proteins (oxidizers, detergents), instability (e.g., latent aldehydes), and lack of druggability (e.g., compounds lacking both oxygen and nitrogen). The Lilly’s MedChem rules have been implemented in the https://github.com/IanAWatson/Lilly-Medchem-Rules code, which is written in ruby. The PyMolGen code interacts with the ruby code to filter molecules as they are being created; or they can be filtered once the generation run is finished.

## Results and Discussion

The ChEMBL database was fragmented following the fragmentation rules and 16,309 fragments were obtained. A clear difference in the frequencies of derived fragments was observed (Figure 7), where the most prevalent fragments are exponentially more common than the rest.

**Figure 7:**
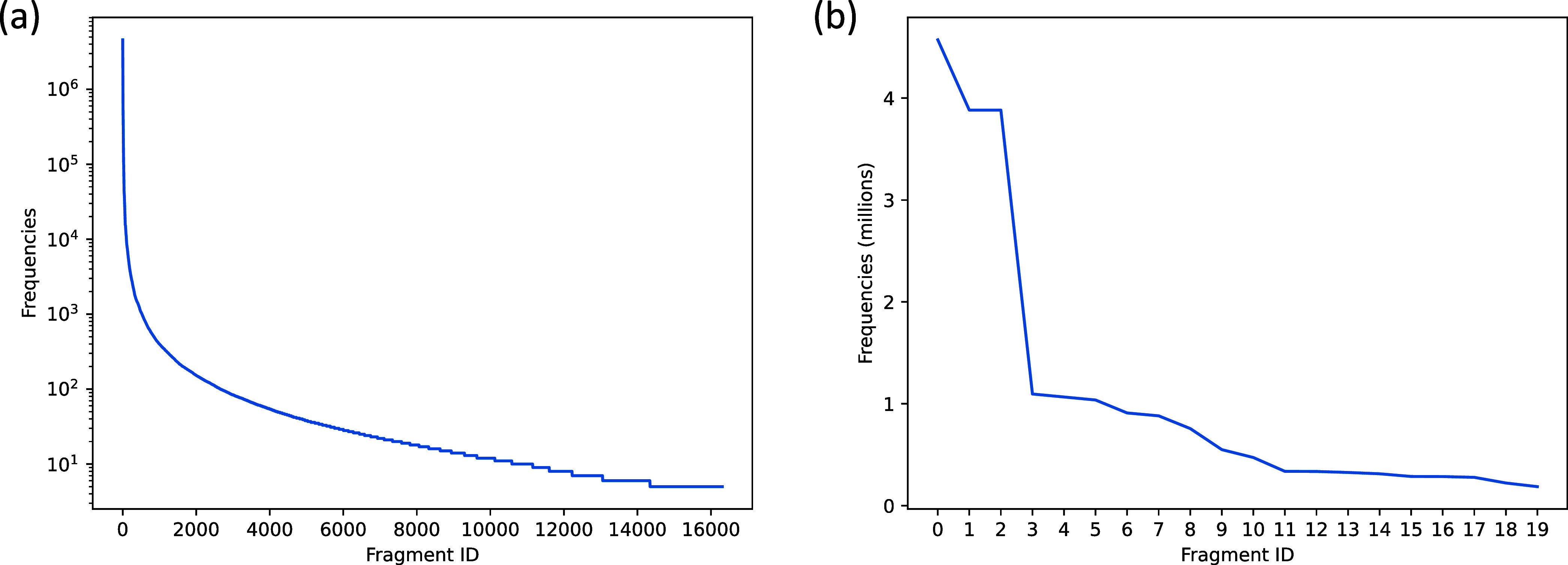
Frequencies of fragments observed in the ChEMBL database: (a) all fragments, (b) top 20 most frequent fragments. The simplest fragments, shown in Figure 8, dominate the distribution.

The top 20 most frequent fragments (Figure 8) consist of very simple structures that are ubiquitous in drug-like compounds, such as CH_2_, CH_3_ and amide groups. As all fragments are treated with explicit hydrogen atoms, different hydrogen substitution patterns give different fragments, as is observed in Figure 9 with CH_2_ versus CH_3_, O versus OH, N versus NH versus NH_2_, or the various substitution patterns of benzene rings.

**Figure 8:**
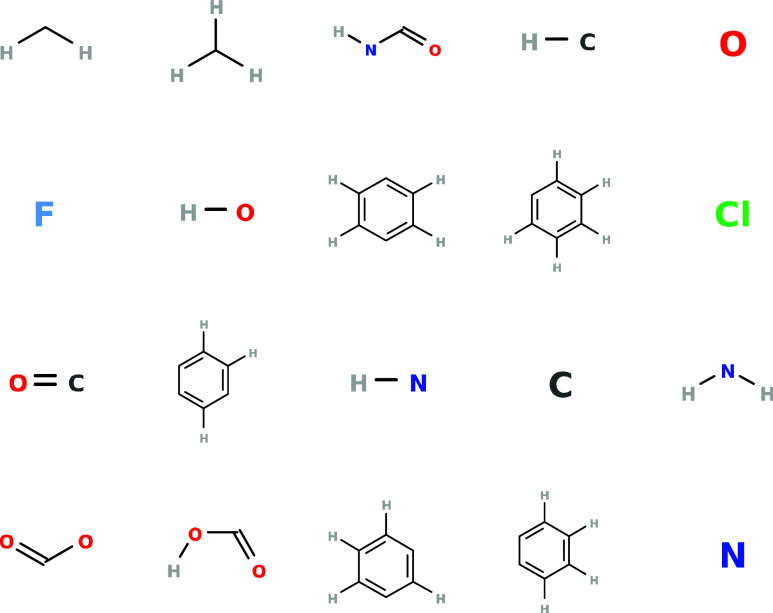
Top 20 most frequent fragments obtained from the ChEMBL database; explicit hydrogen atoms are shown. Missing hydrogen atoms indicate available attachment points to form single bonds with other fragments, following combination rules.

**Figure 9:**
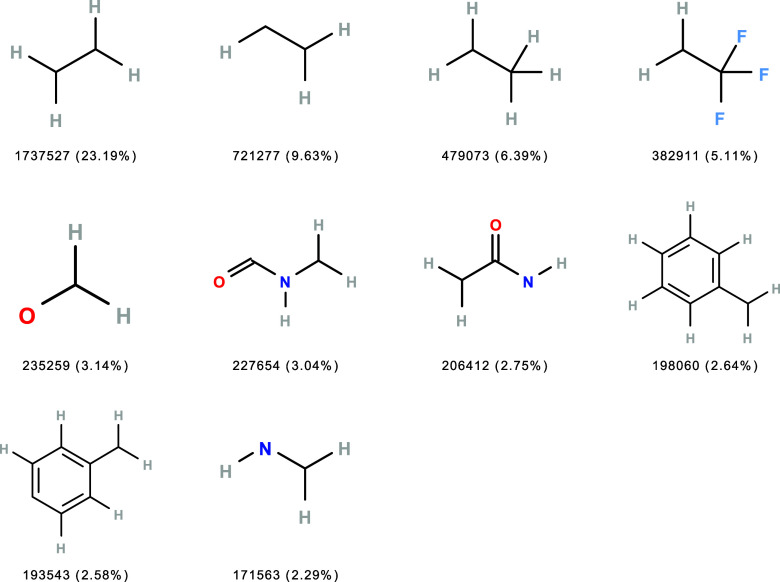
Most likely bonds to the CH_2_ fragment and their frequencies shown in absolute and in relative terms (as a proportion of the global distribution of connectivity to fragment CH_2_).

The combination rules stored in the (***i***, ***j***, ***k***, ***l***) bond vectors are obtained through the fragmentation of the compounds in a database. Figure 9 shows the ten most frequent bond vectors for the CH_2_ fragment. The most frequent bond is with another CH_2_ fragment, which is a very common pattern in drug-like compounds as alkyl chains are reconstructed. Similar to the distribution of fragment frequencies, the most likely bonds dominate the frequency distribution. The fragments were further filtered to remove unwanted structures (Figure 4), as discussed in the Methods section.

### Random Generation of 138 Million Molecules

A model run was performed from the phenylisoxazole parent compound with functionalization at the *para* and *meta* positions. 138 million distinct molecules were generated and activities against BRD4 were predicted using a machine-learning QSAR model. The distribution of predicted activities is shown in Figure S1. The physicochemical properties relevant for drug design (MW, number of H-bond donors, H-bond acceptors, rotatable bonds, log *P* and PSA) were calculated (Figure S2) and they show a smooth distribution within the permitted values, according to the extended Lipinski rules.

### Systematic Generation with the Fragment Graph Approach

An exhaustive generation of molecules from the phenylisoxazole parent structure was subsequently carried out with derivatization permitted at the *meta* and *para* positions as before, with −10 as the threshold for the log of the build probability (Figure 10), and no imposition of a *depth* condition. The total number of unique molecules generated was 298 million. Initially, the output volume increases as new fragments are added, and the combinatorial possibilities of fragment arrangement increases, exacerbated by increased branching. However, beyond addition of five fragments to the core structure, the number of output molecules decreases, since the increasing complexity decreases the likelihood of the build probability threshold being met. With the threshold as implemented here, no further molecules can be built beyond a +15 addition and convergence is reached.

**Figure 10:**
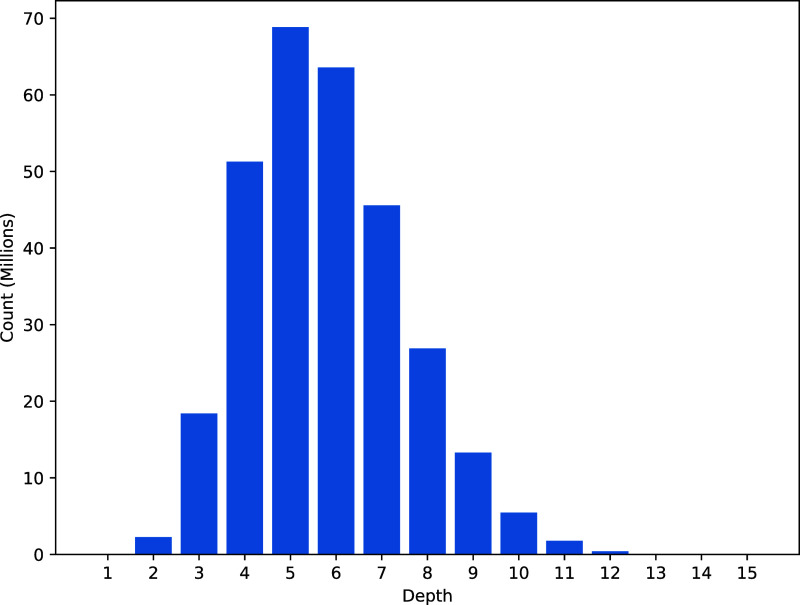
Exhaustive generation of molecules from the phenylisoxazole parent structure with substitutions at the *meta* and *para* positions, with a 10^–10^ for the build probability threshold. The generation converges after the addition of 15 fragments, and the number of possible molecules reaches a maximum at +5 fragments.

### Random Generation of 2.1 Billion Molecules with the Fragment Graph Approach

Taking advantage of the enhancements in the graph-based approach, a large-scale random generation from the phenylisoxazole parent structure was carried out, with substitutions at the *para* and *meta* positions, as described in Methods (Figure 11). 2.1 billion unique molecules were obtained in the 100 24 h parallel runs compared to 138 million unique molecules from 100 72 h parallel runs with the first version of PyMolGen. Molecules were saved from the addition of 3 fragments upward (depth +3) up to depth 12. Molecules at depths 1 and 2 were not saved to avoid the generation of large intermediate files and the slow-down of the code. These molecules can, however, be generated using the systematic code, as it was estimated using the count option of the molecular generator to be less than 70 million molecules.

**Figure 11:**
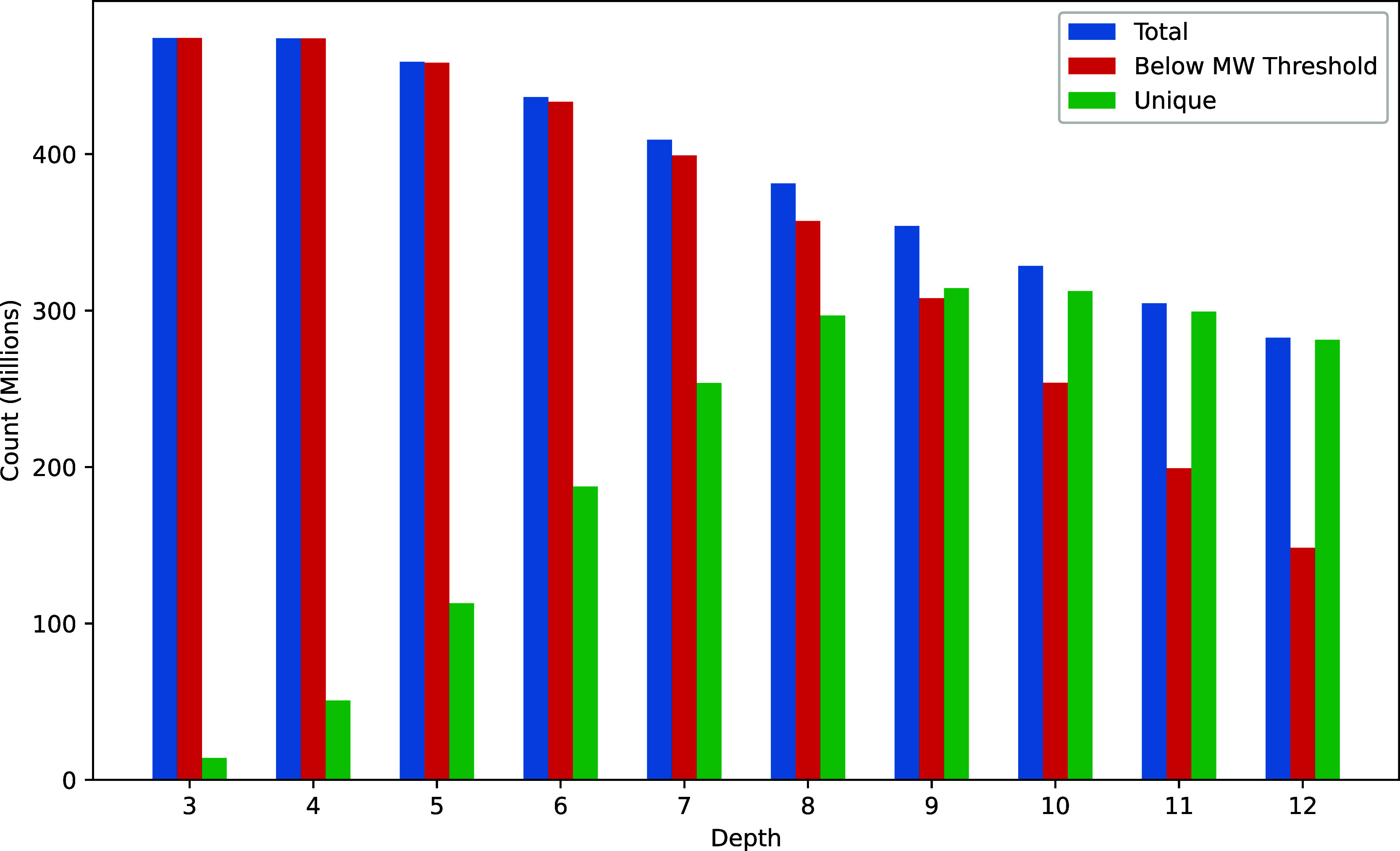
Random generation of molecules from the phenylisoxazole parent structure with substitutions at the *meta* and *para* positions using the graph-based approach. 3.9 billion molecules were generated, with 2.1 billion unique molecules, and 3.5 billion nonunique molecules within a molecular weight threshold of 500 Da.

The distribution of molecules generated versus depth is shown in Figure 11. Several conclusions can be drawn from it. First, the number of unique molecules out of those generated is a small fraction at low depths, with, for example, only 3% unique molecules at depth 3. This proportion increases with the complexity of the molecules, as the addition of further fragments introduces further attachment points and possibilities for branching and isomerism. From depth 7 onward, most molecules made are distinct, comprising more than 95% at depth 10. With regards to the molecular weight of the generated molecules, most molecules (87%) up to depth 9 fall within the 500 Da threshold.

The distributions of build probabilities and molecular weights were analyzed for one of the 100 parallel random generations, for a total of 48.8 million nonunique molecules (Figure S3). These results were consistent with those from the other PyMolGen generations and provide a rationalization of the results observed in the random generation using bond probabilities and systematic generation described before. As can be observed in the distribution of build probabilities, the average of the log of build probabilities is below −10 at a depth of 3 and keeps gradually decreasing upon the addition of further fragments, reaching a value of close to −55 at depth 12. This is consistent with the results observed when comparing the random generation with the systematic generation, where out of the 150 million random compounds made, only 50 of those were present in the 300 million from the systematic generation. In that systematic generation a threshold of −10 was chosen for the log of the build probability; so, it is indeed logical that the random generation made several compounds below that threshold. This analysis also explains why after an addition of seven fragments (from the second random generation) the majority of molecules are not repeated, as the chemical space becomes enormous and it is extremely unlikely a molecule would be repeatedly generated. In terms of molecular weight, the results in Figure S3 complement those in Figure 11, as from depth 9 but especially depth 10, a significant number of molecules are above a weight threshold of 500 Da.

With these results in mind, a user of PyMolGen can decide on what the optimal implementation of the methodology will be according to the specific needs of their project. Either a random or a systematic generation can be selected and the use of a molecular weight threshold prevents undesirable molecule generation at higher depths.

PyMolGen has been used as the molecular generation method in a recent study,^34^ which introduced SimDMTA, an in-silico framework designed to simulate the Design-Make-Test-Analyze (DMTA) cycle used in preclinical drug discovery. This work showed that uncertainty-based sampling outperforms greedy and hybrid approaches in hit discovery and in addition provides better generalization in the prediction of the docking scores by the machine-learning based model. PyMolGen has been extensively applied in BRD4 drug-discovery projects and these results will be shared in upcoming publications. One application with some associated experimental data is described below, in order to illustrate the approach.

The output from a consideration of simultaneously *meta*/*para*-substituted vectors (see the second step of Figure 5) was clustered based on scaffold analysis.^35^ This identified several decorated bicyclic scaffolds, comprising a mixture of previously reported Bromodomain and Extra-Terminal (BET) inhibitors from the literature, e.g., benzimidazolone (**1**) (Figure 12), and novel lead-like motifs.^36–38^

**Figure 12:**
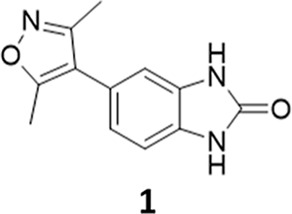
Structure of benzimidazolone, **1**, one of several cores generated from a focused PyMolGen run to identify bicyclic scaffolds for targeted BET inhibition.

Compound **1** had a predicted pIC_50_ of 5.4. Subsequent experimental measurement of potency against BD1 of BRD4, as determined by TR-FRET binding assays (*n* = 2), gave a pIC_50_ of 5.7. Docking of **1** into the BRD4-BD1 binding pocket was carried out using the OEDocking POSIT program to assess the relevant interactions (Figure 13).^39^ This confirmed that the isoxazole moiety in **1** functions as an *N*-acetylated lysine mimetic and the WPF shelf can be accessed through *N*-functionalization.^40,41^ The modeling indicated that the imidazolone nitrogen on the same face of the molecule as the isoxazole (*N*-1) was directed toward the WPF shelf. This formed the basis of a hypothesis that substitution at this position would likely produce more potent analogues compared to substitution at the other nitrogen (*N*-3).

**Figure 13:**
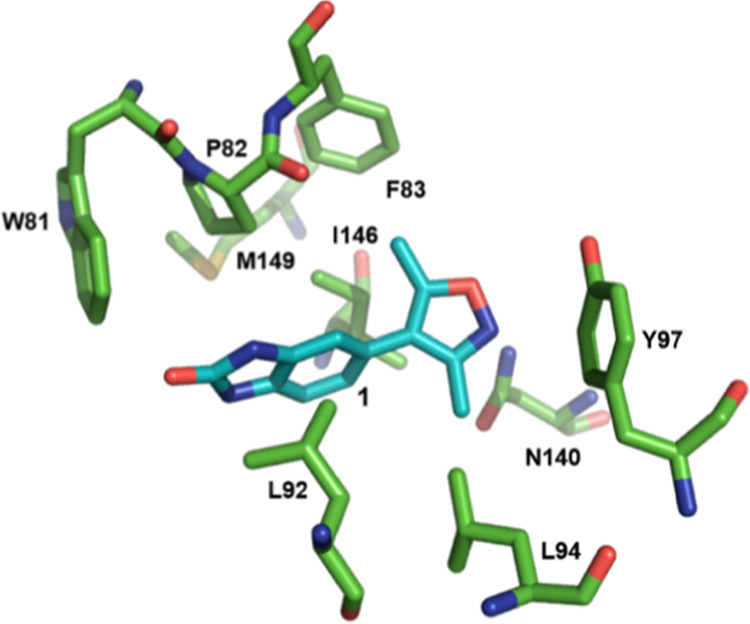
Binding mode of **1** in BD1 of BRD4. Amino acids (green) and **1** (teal) are displayed as stick models. Oxygen atoms are shown in red, nitrogen in blue and sulfur in yellow. Key interaction occurs between isoxazole and asparagine residue (N140) in the *N*-acetylated lysine binding pocket. Docking analysis carried out using the OEDocking POSIT program and the figure generated was in PyMOL using PDB 5S9R.^39,42^

PyMolGen was used to explore disubstitution of the benzimidazolone scaffold. The objective was to ensure output compounds were within an acceptable physiochemical space. Therefore, limits were set for appropriate physicochemical parameters: i.e., PFI <8, MW <500 g mol^–1^, 0.5 ≤ cLogP ≤5, HBD ≤5, HBA ≤10, #rotatable bonds <7 and PSA ≤140 Å. Approximately 13,000 molecules satisfied these criteria. The top 100 in terms of predicted potency were selected. They all had predicted pIC_50_ values in the range of 6.8 to 7.1 and predicted log *D* values in the range of 0.8–3.0. The predictions provided sufficient encouragement for further evaluation of these outputs in terms of synthetic tractability, which yielded alcohol (**2**) and sulfonamide (**3**) as the two most viable targets for initial synthesis (Figure 14). These were both predicted to have good potency (IC_50_ ∼200 nM) and an excellent physicochemical profile (PFI <6) according to the model, while also satisfying all the designated property limits and necessary filters.^29^

**Figure 14:**
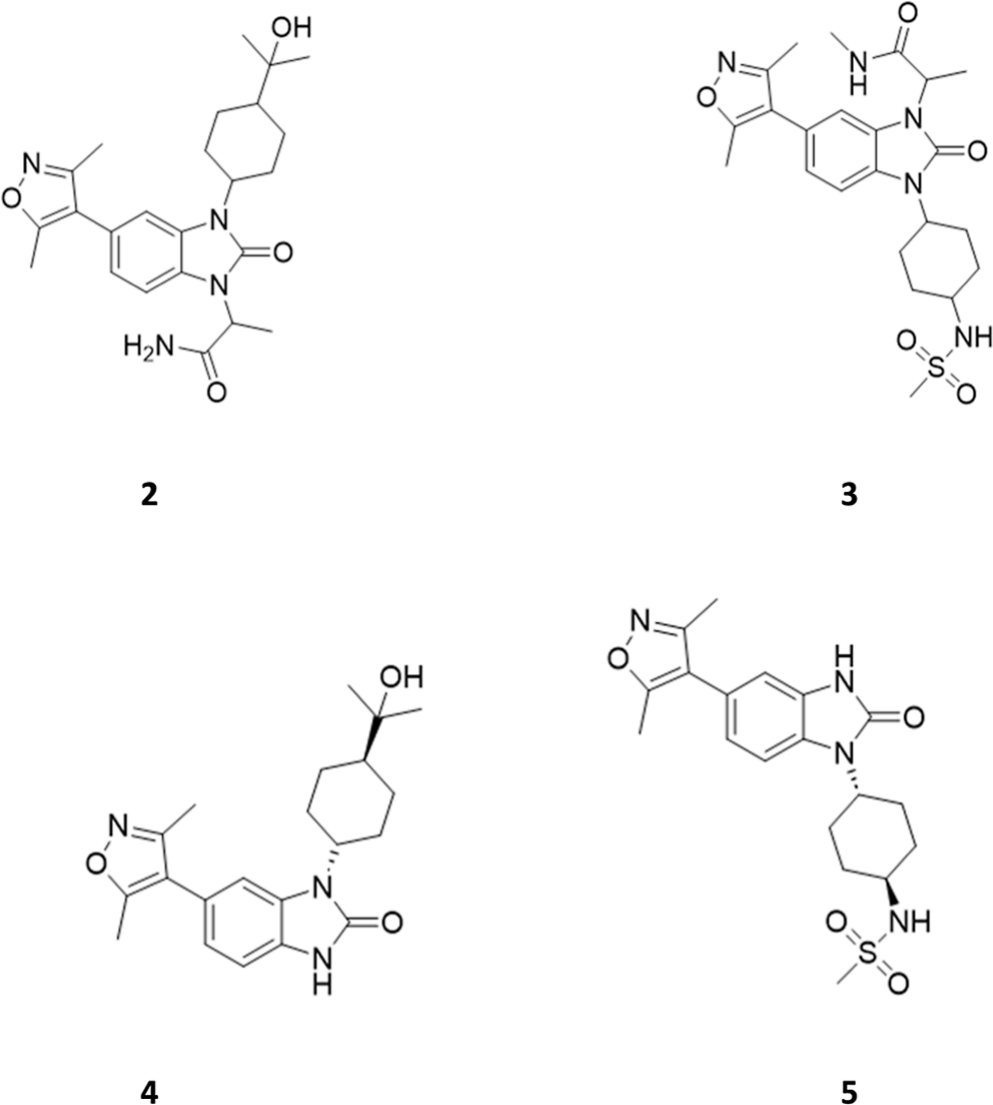
Structures of novel BET inhibitors **2** and **3**, both PyMolGen outputs, and their respective precursors, **4** and **5**. The properties of the compounds are as follows. For **2**: predicted pIC_50_ = 6.8; predicted PFI = 5.9; MW = 440.5 g/mol; HBD = 2; HBA = 8; number of rotatable bonds = 5; PSA = 113 Å. For **3**: predicted pIC_50_ = 6.7; predicted PFI = 6.0; MW = 489.6 g/mol; HBD = 2; HBA = 10; number of rotatable bonds = 5; PSA = 133 Å.

*In silico* docking of **2** and **3** using the OEDocking POSIT program was consistent with the earlier pose generated for compound **1**, which indicated that substituents at the *N*-1 position in this scaffold would likely engage with the WPF shelf and substituents at the *N*-3 position would be directed toward solvent. There is literature precedent for the use of cyclohexyl substituents as nonaromatic bioisosteres to probe the WPF shelf in small molecule BET inhibitors, supporting the basis for selection of this functional group by the PyMolGen.^43^ While cyclohexyl substituents are well tolerated from a potency perspective, their incorporation can increase lipophilicity and reduce metabolic stability, which could well be mitigated by the addition of the tertiary alcohol in **2** and the methyl sulfonamide in **3**.^44^ With the *N*-3 substituent being directed toward solvent, the carboxamide in **2** and sulfonamide in **3** would most likely act to tune global lipophilicity due to their hydrogen bonding capabilities.^45^ Compounds **2** and **3** and their precursors **4** and **5** (Figure 14) were biologically evaluated (Table 1).

**Table 1:** Predicted and Experimental Biological Data for PyMolGen Targets, **2** and **3**, and Intermediates, **4** and **5** ^a^ ^,^ ^24^

Compound	Pred. pIC_50_	Exp. pIC_50_	Pred. PFI	Exp. PFI
**2**	6.5	5.6	5.8	6.2
**3**	6.5	5.6	5.8	5.9
**4**	6.4	6.3	6.8	6.4
**5**	6.3	5.6	6.4	5.8

a Experimental potency against BD1 of BRD4 was determined by TR-FRET binding assays (*n* = 2).

The alcohol-containing intermediate **4** was the most potent analogue (pIC_50_ = 6.3), providing preliminary support of the docking hypothesis that the *N*-1 substituent is engaging with the WPF shelf. While *N*-alkylation of **4** was not predicted to increase potency significantly given that docking suggests that the *N*-3 substituent extends toward solvent, it was surprising that this was accompanied by a decrease in potency in the fully elaborated analog **2** and only a slight reduction in PFI (0.2 log units) compared to **4**. While the bicyclic benzimidazoline series displayed moderate potency and reasonable physiochemical profiles, other templates identified were more promising in terms of overall developability and these will be reported in future publications.

## Conclusions

We have devised a molecular generation algorithm that derives the combination rules and frequencies between fragments from a database—in this case, we have used the ChEMBL database and provides a mechanism for optimization of a hit series on defined, synthetically tractable vectors. The algorithm constructs molecules in a manner that reflects the relationships between fragments in the underlying database, poising the generated molecules to comprise privileged extended substructures and link fragments in a manner that are more likely to be synthetically tractable. Different generation modes are available: molecules can be generated from random sampling of the underlying distribution or in a systematic fashion. The latter introduces the concept of a build probability, which scores the generated molecules according to an estimation of the likelihood of their generation through a hypothetical exhaustive enumeration.

We have applied the developed methods to the generation of BRD4 inhibitors from a weakly potent parent structure and have scored the generated molecules using an in-house 2D-QSAR model after having filtered the generated molecules based on structural properties and the Eli Lilly’s MedChem rules. We have generated 138 million and 2.1 billion molecules following the two random generation algorithms, which use an all-atom and a fragment graph-based approach, respectively, and have generated 300 million molecules in a systematic fashion, with a build probability threshold of 10^–10^.

The algorithm can be applied to drug discovery projects where a hit or lead molecule is available, by applying the combination rules obtained from ChEMBL or by deriving new combination rules from another database, which can be done with the PyMolGen code. The generation is highly customizable as the attachment points of the parent structure, the maximum molecular weight and number of fragments added in the final molecules can be configured. The use of a build probability threshold allows for convergence in the generation of molecules, so that all derivatives can be explicitly enumerated.

## Supplementary Material



## Data Availability

The software described in this paper is available from the GitHub repository: https://github.com/HirstGroup/PyMolGen.

## Notes

Figures showing distributions of activities and properties; additional experimental details of the synthesis and characterization of the new compounds (PDF)
