# Supplementary material for: PyMolGen: Database-Driven Molecular Generation of Drug-Like Compounds
Source: J Chem Inf Model. 2026 Jun 16;66(15):9188–99. doi: 10.1021/acs.jcim.6c00689 (PMC13471430; doi:10.1021/acs.jcim.6c00689)
Supplement: Supplementary file 1 [file ci6c00689_si_001.pdf]

Supporting Information

PyMolGen: Database-Driven Molecular Generation of Drug-Like Compounds

Bruno N. Falcone,[a] Michael J. Hutcheon,[a] Jaffer M. Zaidi,[b] Michael J. Stocks,[b] Kirsty E. Hamill,[c] Craig Jamieson,[c] Huw J. Williams,[c] David S. Palmer,[c] Andrew Baxter,[d] Stephen D. Pickett[e] and Jonathan D. Hirst[a*]

[a]School of Chemistry, University of Nottingham, University Park, Nottingham NG7 2RD, United Kingdom

[b]Biodiscovery Institute, School of Pharmacy, University of Nottingham, University Park, Nottingham NG7 2RD, United Kingdom

[c]Department of Pure and Applied Chemistry, University of Strathclyde, Glasgow G1 1XL, United Kingdom

[d]Medicines Research Centre, GSK, Stevenage SG1 2NY, United Kingdom

[e]Department of Cheminformatics, Research Technologies, GSK, Stevenage SG1 2NY, United Kingdom

[*]jonathan.hirst@nottingham.ac.uk

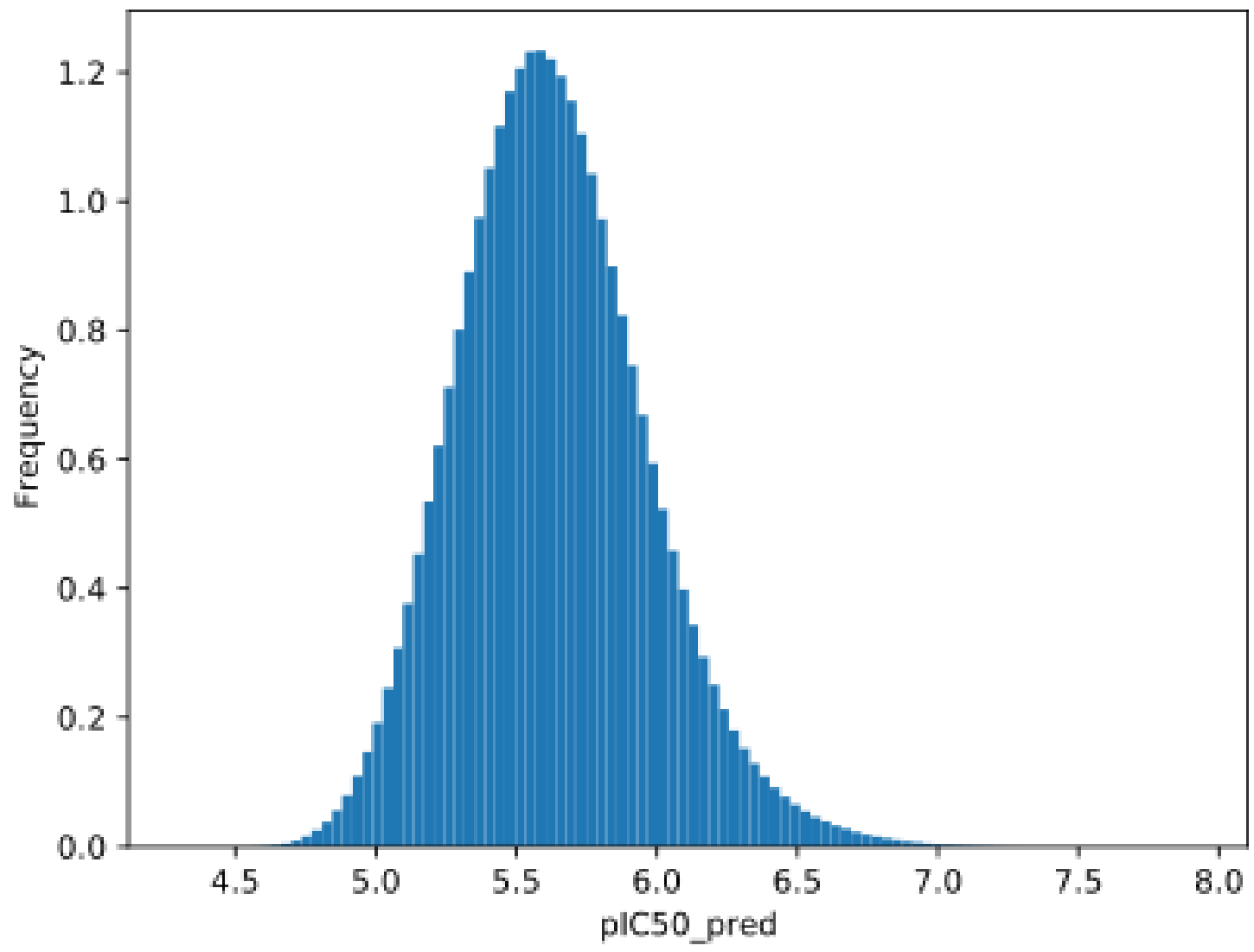

Figure S1. Distribution of predicted pIC_{50} values against BRD4 for molecules generated from phenyl dimethylisoxazole at the *meta*, *para*, and *meta*-*para* positions.

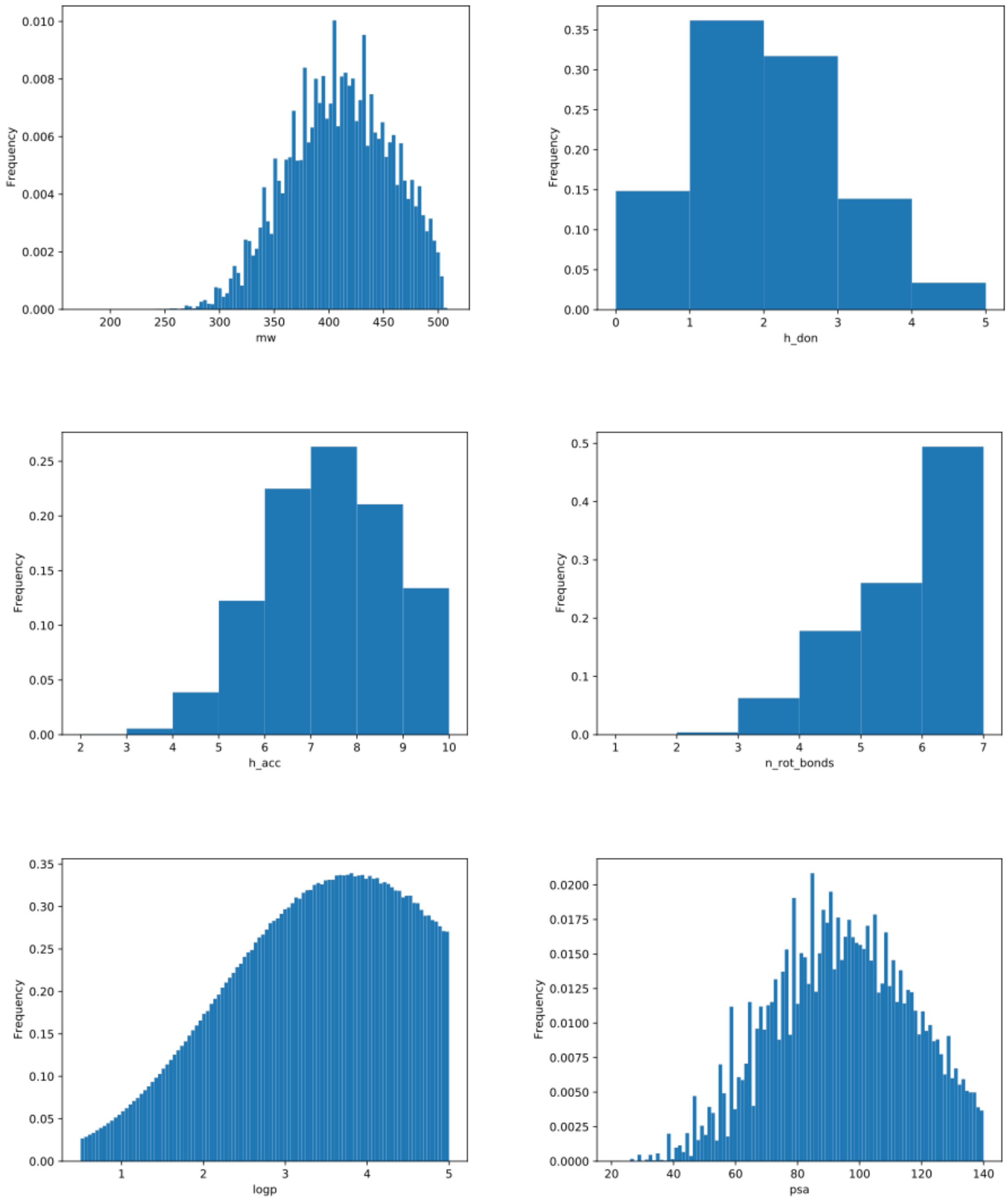

Figure S2. Distribution of MW, number of H-bond donors, H-bond acceptors, rotatable bonds, log *P* and PSA within the molecules generated from the phenylisoxazole parent structure.

(a)

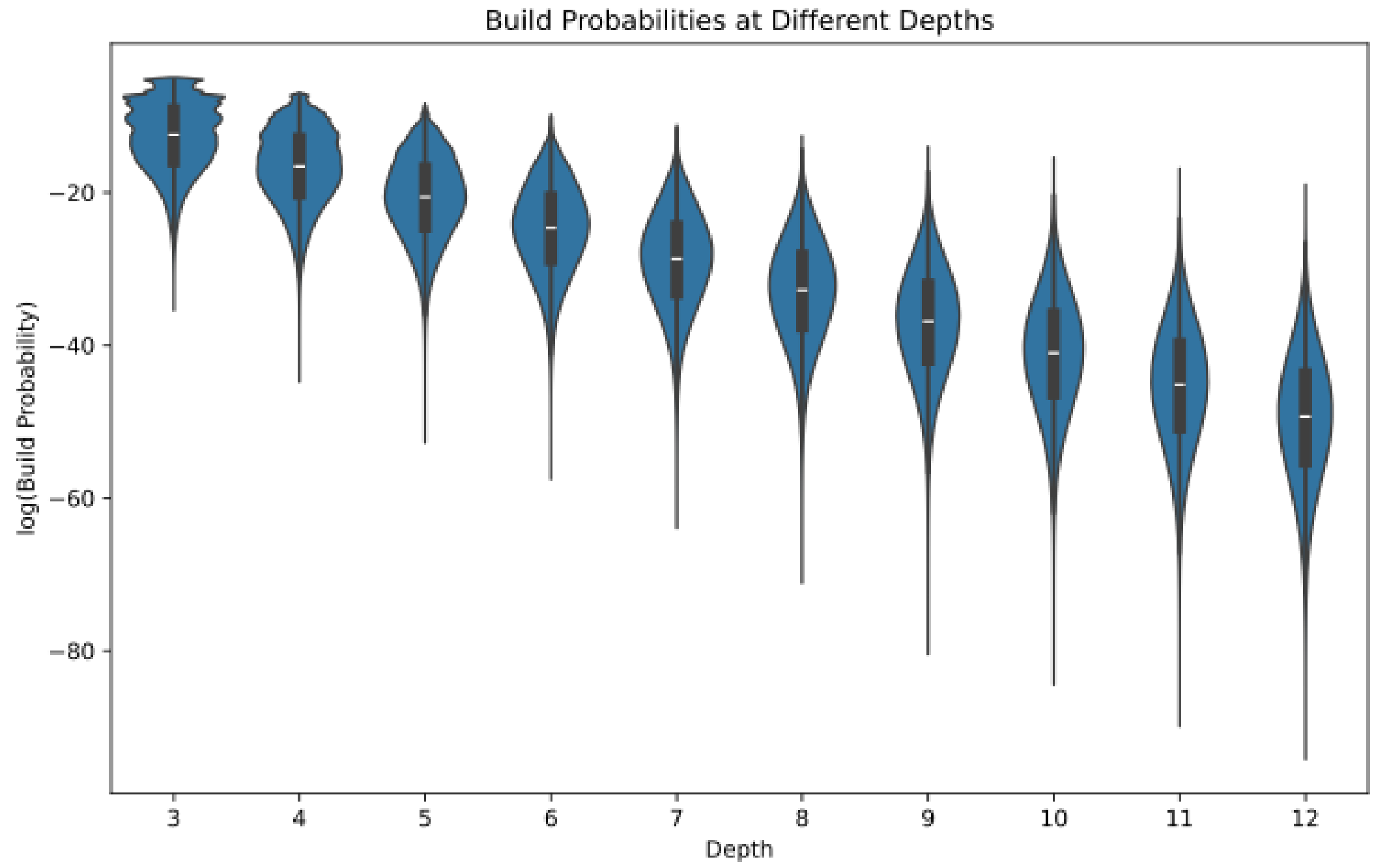

(b)

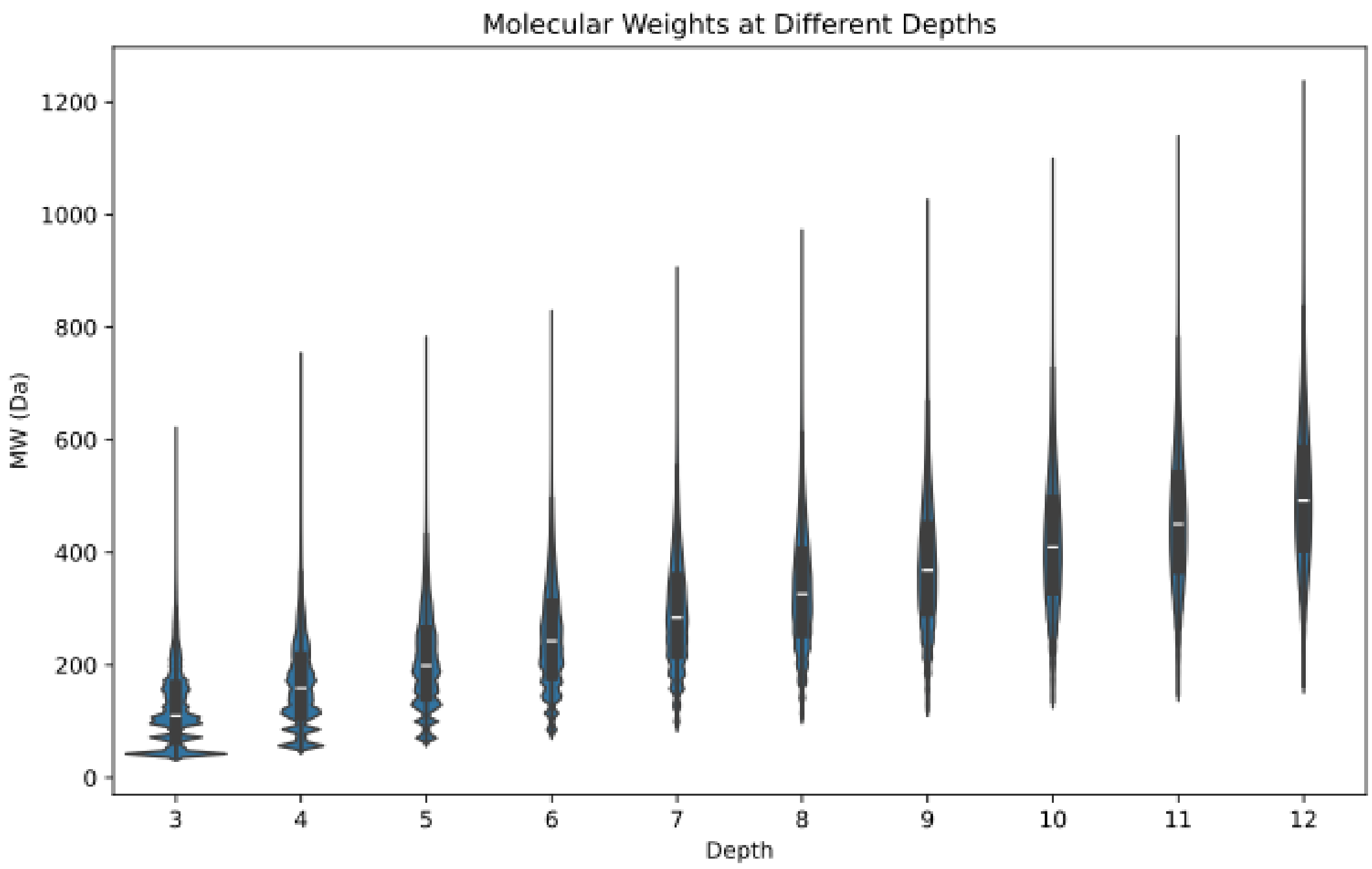

Figure S3. Violin plots showing the distribution of (a) build probabilities and (b) molecular weights at each depth of the molecules generated in a representative random run.

Experimental chemistry

General Details

Molecular modelling studies were conducted using the OpenEye Make Receptor and OpenEye OEDocking POSIT programs for docking and the OpenEye VIDA and Schrödinger PyMOL programs for visualisation.[1] All programs were used with the default parameters. No unexpected or unusually high safety hazards were encountered. Unless otherwise stated, all reactions were carried out under an atmosphere of nitrogen in conventional glassware. Cooling to 0 °C was effected using an ice-water bath. All water was deionised before use. Chemicals and solvents of analytical and HPLC grade were purchased from commercial suppliers and used without further purification. Anhydrous solvents were obtained from Merck and transferred from the Sure Seal bottles by syringe under an atmosphere of nitrogen using standard Schlenk techniques. Room temperature varied between 18 °C and 25 °C. All reported compounds are >95% pure. The purity assessments were made using the UV absorption spectra collected as part of LCMS analysis and/or NMR. Analytical TLC was undertaken on Merck aluminium-backed silica gel 60 F_{254} plates. Developed TLC plates were visualised by irradiation with UV light (254 nm). LCMS data was obtained on a Shimadzu UFLCXR HPLC system coupled to an Applied Biosystems API 2000 LC/MS/MS with ESI, using a Phenomenex Gemini-NX C18 110 Å column (50 mm x 2 mm x 3 µm) at a flow rate of 0.5 mL/min. t_R is reported using a gradient method of 95:5-2:98, A:B over either 5 min (Method 1) or 13 min (Method 2). Solvent A is 0.1% formic acid in H_2O and solvent B is 0.1% formic acid in MeCN. HRMS data was acquired on a Bruker MicrOTOF II ESI-TOF instrument. Automated column chromatography was executed on a Biotage HPFC SP4 system for normal-phase purification, using silica HP (50 µm) cartridges, and an Interchim PuriFlash 4100 system for reverse-phase purification, using silica C18-HP (30 µm) cartridges. NMR spectra were recorded at 298 K on a Bruker AV spectrometer operating at the nominal ^{1}H and ^{13}C frequencies stated. All NMR data was processed using Mestrelab Research MestReNova software. Chemical shifts (δ) are given in ppm and are referenced to residual solvent peaks for ^{1}H and ^{13}C{^{1}H} respectively as appropriate for the NMR solvent used to obtain the spectra. Coupling constants (*J*) are given in Hz. The multiplicity of a ^{1}H or ^{13}C NMR signal is designated by standard abbreviations as defined by The ACS Guide to Scholarly Communication.[2] All property evaluation was performed by colleagues in the Discovery Biology Screening and Molecular Modalities Capabilities departments at GSK. The *in vitro* biological assays used to determine potency against BRD4-BD1 (TR-FRET), hydrophobicity (chrom $LogD_{7.4}$), kinetic solubility (CAD) and membrane permeability (PAMPA) have all been reported previously and hence are not detailed again here.[3]

Synthetic Procedures

*2-((1r,4r)-4-((5-Bromo-2-nitrophenyl)amino)cyclohexyl)propan-2-ol (**S1**)*

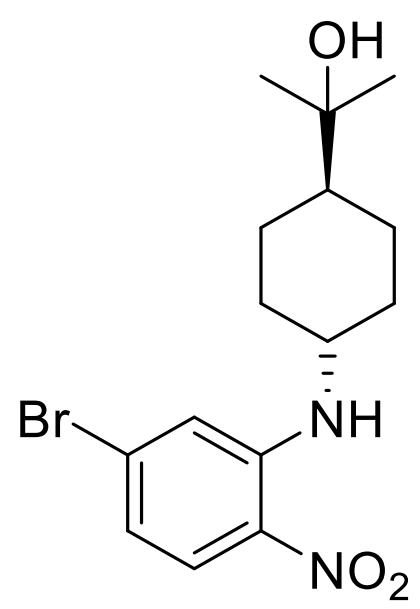

To a solution of 4-bromo-2-fluoro-1-nitrobenzene (700 mg, 3.18 mmol) in DMF (16 mL) was added 2-(*trans*-4-aminocyclohexyl)propan-2-ol (500 mg, 3.18 mmol, 1.00 eq) and K_2CO_3 (829 mg, 6.36 mmol, 2.00 eq) and the reaction was heated at 70 °C for 2 h. The reaction mixture was cooled to rt and diluted with H_2O (30 mL). The resulting precipitate was filtered, washed with H_2O and dried under vacuum to yield **S1** as a yellow solid (1.02 g, 89%). ^{1}H NMR (400 MHz, DMSO-d$_6$) δ: 7.97 (1H, d, *J* = 7.8 Hz), 7.91 (1H, d, *J* = 7.9 Hz), 7.35 (1H, s), 6.81 (1H, d, *J* = 9.2 Hz), 4.08 (1H, s), 3.67-3.52 (1H, m), 2.11-1.97 (2H, m), 1.91-1.75 (2H, m), 1.36-1.13 (5H, m), 1.05 (6H, s). ^{13}C{^{1}H} NMR (101 MHz, DMSO-d$_6$) δ: 144.9, 131.2, 129.9, 128.2, 118.1, 116.9, 70.5, 51.0, 47.7, 32.3, 27.0, 25.6. LCMS (ESI$^+$): $C_{15}H_{21}BrN_2O_3$ $[M+H]^+$ m/z calcd 357.1; found 357.0. t_R (Method 1): 2.91 min.

*2-((1r,4r)-4-((5-(3,5-Dimethylisoxazol-4-yl)-2-nitrophenyl)amino)cyclohexyl)propan-2-ol (**S2**)*

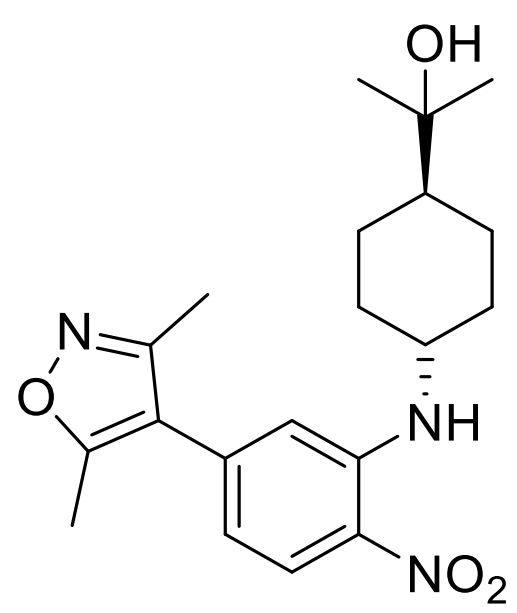

To a solution of **S1** (1.01 g, 2.83 mmol) in dioxane (24 mL) and H_2O (5.9 mL) was added 3,5-dimethylisoxazole-4-boronic acid (1.20 g, 8.48 mmol, 3.00 eq), Na_2CO_3 (599 mg, 5.65 mmol, 2.00 eq) and $Pd(PPh_3)_4$ (327 mg, 283 µmol, 0.100 eq) and the reaction was heated at reflux for 2 h. The reaction mixture was cooled to rt, eluted through a plug of Celite with DCM and concentrated under reduced pressure. The crude material was purified by reverse-phase column chromatography (80:20-0:100, H_2O:MeCN) to yield **S2** as an orange solid (640 mg, 60%).^{1}H NMR (400 MHz, DMSO-d$_6$) δ: 8.12 (1H, d, *J* = 8.9 Hz), 7.96 (1H, d, *J* = 7.8 Hz), 7.06 (1H, s), 6.69 (1H, d, *J* = 8.9 Hz), 4.07 (1H, s), 3.70-3.53 (1H, m), 2.45 (3H, s), 2.27 (3H, s), 2.15-2.03 (2H, m), 1.88-1.78 (2H, m), 1.34-1.16 (5H, m), 1.05 (6H, s). ^{13}C{^{1}H} NMR (101 MHz, DMSO-d$_6$) δ: 166.5, 158.0, 144.5, 138.4, 129.7, 127.0, 115.8, 115.1, 114.7, 70.5, 50.9, 47.8, 32.6, 27.1,

25.6, 11.6, 10.6. LCMS (ESI$^+$): $C_{20}H_{27}N_3O_4$ $[M+H]^+$ m/z calcd 374.2; found 374.2. t_R (Method 1): 2.82 min.

*2-((1r,4r)-4-((2-Amino-5-(3,5-dimethylisoxazol-4-yl)phenyl)amino)cyclohexyl)propan-2-ol (**S3**)*

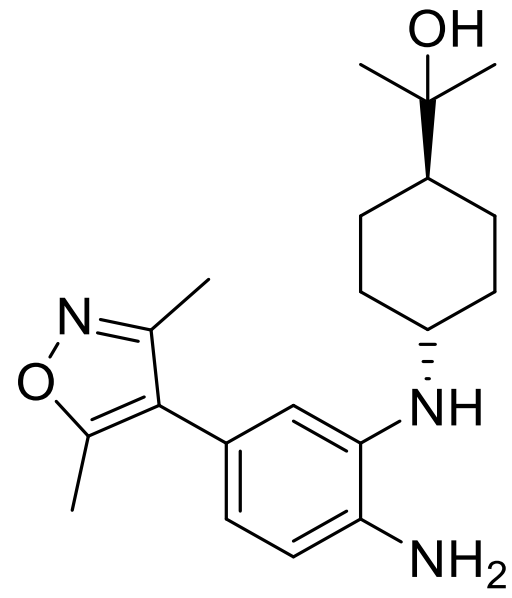

To a solution of **S2** (618 mg, 1.65 mmol) in MeOH (22 mL) and H_2O (2.6 mL) was added Zn (863 mg, 13.2 mmol, 8.00 eq) and NH_4Cl (115 mg, 2.15 mmol, 1.30 eq) and the reaction was heated at reflux for 4 h. The reaction mixture was cooled to rt, eluted through a plug of Celite with DCM and concentrated under reduced pressure. The crude material was purified by reverse-phase column chromatography (90:10-10:90, H_2O:MeCN) to yield **S3** as an off-white solid (284 mg, 50%). ^{1}H NMR (400 MHz, DMSO-d_6) δ: 6.59 (1H, d, *J* = 8.3 Hz), 6.39-6.30 (2H, m), 4.65 (2H, s), 4.23 (1H, d, *J* = 7.6 Hz), 4.01 (1H, s), 3.20-3.04 (1H, m), 2.34 (3H, s), 2.17 (3H, s), 2.12-2.02 (2H, m), 1.88-1.75 (2H, m), 1.27-1.08 (5H, m), 1.04 (6H, s). ^{13}C{^{1}H} NMR (101 MHz, DMSO-d_6) δ: 163.5, 158.3, 134.9, 134.7, 118.3, 117.1, 117.0, 114.3, 110.9, 70.6, 51.4, 48.5, 33.0, 27.1, 26.0, 11.3, 10.6. LCMS (ESI$^+$): $C_{20}H_{29}N_3O_2$ $[M+H]^+$ m/z calcd 344.2; found 344.2. t_R (Method 1): 2.12 min.

*6-(3,5-Dimethylisoxazol-4-yl)-1-((1r,4r)-4-(2-hydroxypropan-2-yl)cyclohexyl)-1,3-dihydro-2H-benzo[d]imidazol-2-one (**4**)*

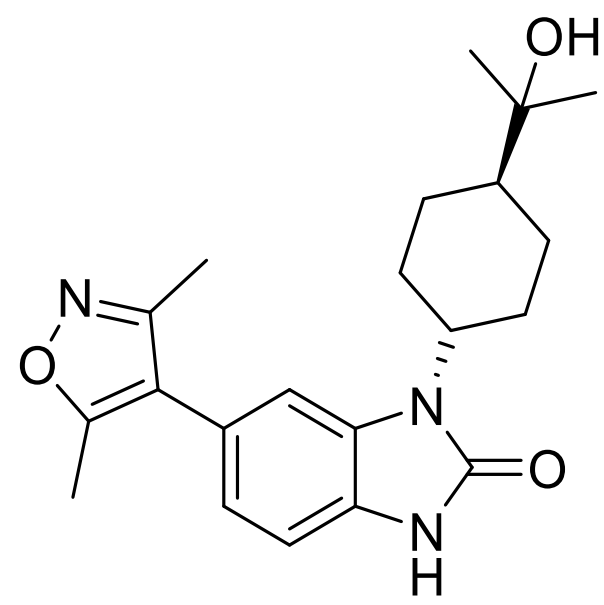

To a solution of **S3** (270 mg, 786 µmol) in DCM (8.8 mL) was added DMAP (19.2 mg, 157 µmol, 0.200 eq) and CDI (255 mg, 1.57 mmol, 2.00 eq) and the reaction was stirred at rt for 3 h. The reaction mixture was concentrated under reduced pressure. The crude material was purified by reverse-phase column chromatography (90:10-10:90, H_2O:MeCN) to yield **4** as a white solid (150 mg, 52%). ^{1}H NMR (400 MHz, DMSO-d_6) δ: 10.90 (1H, s), 7.24 (1H, s), 7.04 (1H, d, *J* = 7.9 Hz), 6.92 (1H, d, *J* = 7.9 Hz), 4.19-4.09 (1H, m), 4.07 (1H, s), 2.38 (3H, s), 2.21 (3H, s), 2.18-2.05 (2H, m), 1.91 (2H, d, *J* = 12.7 Hz), 1.73 (2H, d, *J* = 9.6 Hz), 1.39 (1H, t, *J* = 12.2 Hz), 1.18 (2H, q, *J* = 11.7 Hz), 1.07 (6H, s). ^{13}C{^{1}H} NMR (101 MHz, DMSO-d_6) δ: 164.6, 158.4, 153.9,

129.8, 127.8, 121.9, 121.4, 116.5, 109.4, 108.9, 70.5, 51.9, 47.1, 29.2, 27.1, 26.5, 11.3, 10.5. LCMS (ESI$^+$): $C_{21}H_{27}N_3O_3$ $[M+H]^+$ m/z calcd 370.2; found 370.2. t_R (Method 2): 4.70 min. HRMS (ESI$^+$): $C_{21}H_{27}N_3O_3$ $[M+H]^+$ m/z calcd 370.2126; found 370.2119.

*2-(5-(3,5-Dimethylisoxazol-4-yl)-3-((1r,4r)-4-(2-hydroxypropan-2-yl)cyclohexyl)-2-oxo-2,3-dihydro-1H-benzo[d]imidazol-1-yl)propanamide (**2**)*

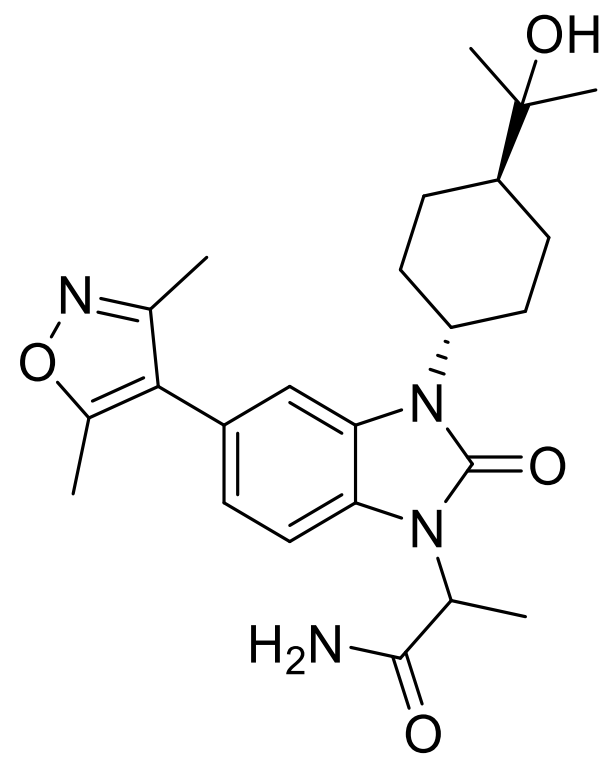

To a solution of **4** (20.0 mg, 54.1 µmol) in DMF (1.0 mL), cooled to 0 °C, was added NaH (60% pure, 2.60 mg, 64.9 µmol, 1.20 eq) and the reaction was stirred for 1 h. 2-Bromopropionamide (10.7 mg, 70.3 µmol, 1.30 eq) was added, the reaction was warmed to rt and stirred for a further 14 h. The reaction mixture was quenched with sat aq NH_4Cl (5.0 mL) and extracted with EtOAc (3 x 5.0 mL). The combined organics were washed with brine (15 mL), dried using a hydrophobic frit and concentrated under reduced pressure. The crude material was purified by reverse-phase column chromatography (90:10-10:90, H_2O:MeCN) to yield **2** as a white solid (10.2 mg, 43%). ^{1}H NMR (400 MHz, DMSO-d_6) δ: 7.54 (1H, s), 7.32 (1H, s), 7.24 (1H, s), 7.14 (1H, d, *J* = 8.0 Hz), 6.98 (1H, d, *J* = 8.0 Hz), 5.01 (1H, q, *J* = 7.3 Hz), 4.19 (1H, t, *J* = 12.1 Hz), 4.08 (1H, s), 2.39 (3H, s), 2.22 (3H, s), 2.20-2.08 (2H, m), 1.92 (2H, d, *J* = 12.6 Hz), 1.78 (2H, d, *J* = 12.1 Hz), 1.53 (3H, d, *J* = 7.3 Hz), 1.40 (1H, t, *J* = 12.1 Hz), 1.20 (2H, q, *J* = 12.6 Hz), 1.07 (6H, s). ^{13}C{^{1}H} NMR (101 MHz, DMSO-d_6) δ: 171.2, 164.7, 158.4, 152.7, 128.9, 127.5, 122.2, 121.2, 116.4, 109.7, 109.5, 70.5, 52.7, 50.3, 47.1, 29.2, 27.1, 26.5, 15.2, 11.3, 10.5. LCMS (ESI$^+$): $C_{24}H_{32}N_4O_4$ $[M+H]^+$ m/z calcd 441.3; found 441.2. t_R (Method 2): 4.60 min. HRMS (ESI$^+$): $C_{24}H_{32}N_4O_4$ $[M+H]^+$ m/z calcd 441.2497; found 441.2494.

*N-((1r,4r)-4-Aminocyclohexyl)methanesulfonamide (**S4**)*

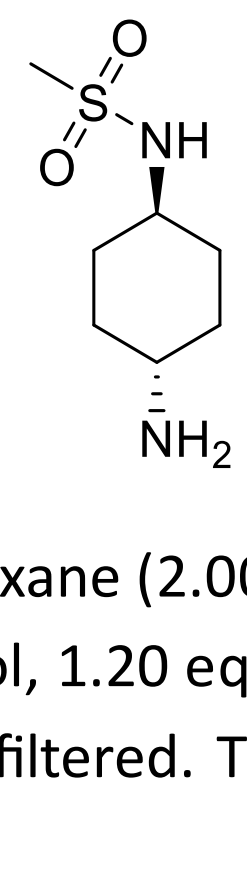

To a solution of trans-1,4-diaminocyclohexane (2.00 g, 17.5 mmol) in DCM (35 mL), cooled to 0 °C was added MsCl (1.63 mL, 21.0 mmol, 1.20 eq) and the reaction was stirred for 1 h. The reaction mixture was warmed to rt and filtered. The resulting precipitate was washed with

DCM and dried under vacuum to yield **S4** as a white solid (3.33 g, 99%). ^{1}H NMR (400 MHz, DMSO-d$_6$) δ: 7.43 (1H, s), 7.00 (2H, s), 3.02 (1H, s), 2.89 (3H, s), 2.83 (1H, s), 2.04-1.77 (4H, m), 1.45-1.14 (4H, m). ^{13}C{^{1}H} NMR (101 MHz, DMSO-d$_6$) δ: 51.1, 48.3, 40.9, 32.3, 29.1. HRMS (ESI$^+$): $C_7H_{16}N_2O_2S$ [M+H]$^+$ m/z calcd 193.1006; found 193.1009.

*N-((1r,4r)-4-((4-(3,5-Dimethylisoxazol-4-yl)-2-nitrophenyl)amino)cyclohexyl)methanesulfonamide (**S5**)*

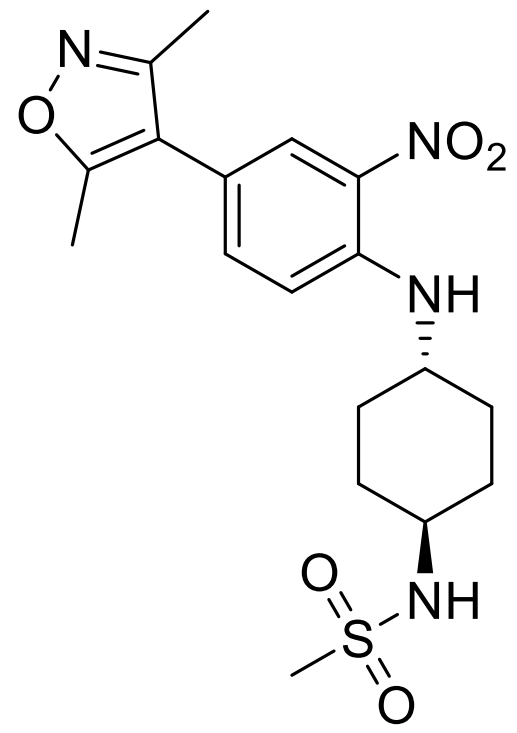

To a solution of 4-bromo-1-fluoro-2-nitrobenzene (625 µL, 5.08 mmol) in DMF (26 mL) was added **S4** (977 mg, 5.08 mmol, 1.00 eq) and K_2CO_3 (1.41 g, 10.2 mmol, 2.00 eq) and the reaction was heated at 70 °C for 17 h. The reaction mixture was cooled to rt and diluted with H_2O (50 mL). The resulting precipitate was filtered, washed with H_2O and dried under vacuum. To a solution of the resulting intermediate in dioxane (43 mL) and H_2O (11 mL) was added 3,5-dimethylisoxazole-4-boronic acid (2.14 g, 15.2 mmol, 3.00 eq), Na_2CO_3 (1.08 g, 10.2 mmol, 2.00 eq) and Pd(PPh$_3$)$_4$ (587 mg, 508 µmol, 0.100 eq) and the reaction was heated at reflux for 22 h. The reaction mixture was cooled to rt, eluted through a plug of Celite with DCM and concentrated under reduced pressure. The crude material was purified by reverse-phase column chromatography (90:10-10:90, H_2O:MeCN) to yield **S5** as an orange solid (239 mg, 11%). ^{1}H NMR (400 MHz, DMSO-d$_6$) δ: 7.99 (1H, s), 7.93 (1H, d, *J* = 7.7 Hz), 7.55 (1H, d, *J* = 9.0 Hz), 7.25 (1H, d, *J* = 9.0 Hz), 7.10 (1H, d, *J* = 7.2 Hz), 3.73-3.51 (1H, m), 3.29-3.11 (1H, m), 2.93 (3H, s), 2.38 (3H, s), 2.20 (3H, s), 2.11-1.90 (4H, m), 1.53-1.36 (4H, m). ^{13}C{^{1}H} NMR (101 MHz, DMSO-d$_6$) δ: 165.2, 158.1, 143.5, 137.1, 130.8, 126.1, 116.6, 115.8, 114.3, 51.2, 49.8, 40.9, 32.0, 30.8, 11.3, 10.4. LCMS (ESI$^+$): $C_{18}H_{24}N_4O_5S$ [M+H]$^+$ m/z calcd 409.2; found 409.1. t_R (Method 1): 2.65 min.

*N-((1r,4r)-4-((2-Amino-4-(3,5-dimethylisoxazol-4-yl)phenyl)amino)cyclohexyl)methanesulfonamide (**S6**)*

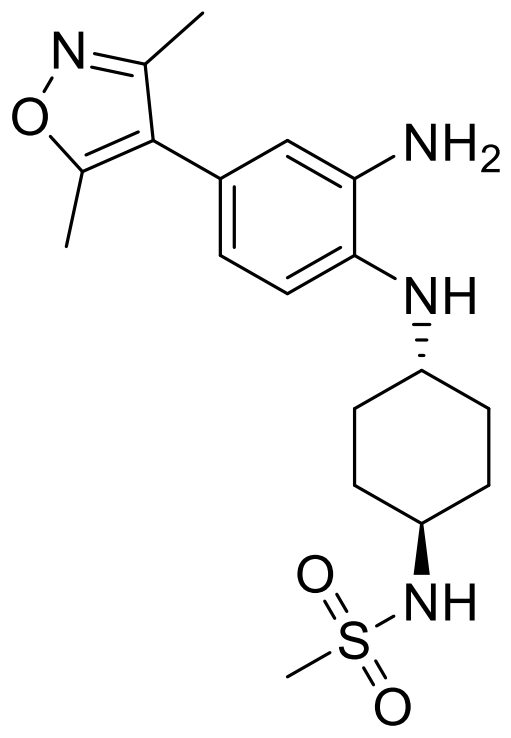

To a solution of **S5** (221 mg, 583 µmol) in MeOH (7.9 mL) and H_2O (930 µL) was added Zn (305 mg, 4.66 mmol, 8.00 eq) and NH_4Cl (40.5 mg, 757 µmol, 1.30 eq) and the reaction was heated at reflux for 22 h. The reaction mixture was cooled to rt, eluted through a plug of Celite with DCM and concentrated under reduced pressure. The crude material was purified by reverse-phase column chromatography (90:10-10:90, H_2O:MeCN) to yield **S6** as a brown solid (94.6 mg, 43%). ^1H NMR (400 MHz, DMSO-d_6) δ: 7.04 (1H, d, *J* = 7.2 Hz), 6.58-6.39 (3H, m), 4.63 (2H, s), 4.29 (1H, d, *J* = 7.5 Hz), 3.23-3.06 (2H, m), 2.92 (3H, s), 2.33 (3H, s), 2.17 (3H, s), 1.99 (4H, dd, *J* = 28.5, 12.4 Hz), 1.31 (4H, dq, *J* = 49.5, 11.7 Hz). $^{13}C\{^1H\}$ NMR (101 MHz, DMSO-d_6) δ: 163.4, 158.2, 135.4, 134.2, 118.1, 117.4, 116.6, 114.6, 110.3, 51.9, 50.3, 41.0, 32.5, 31.4, 11.3, 10.6. LCMS (ESI$^+$): $C_{18}H_{26}N_4O_3S$ $[M+H]^+$ m/z calcd 379.2; found 379.2. t_R (Method 1): 2.02 min.

*N-((1r,4r)-4-(5-(3,5-Dimethylisoxazol-4-yl)-2-oxo-2,3-dihydro-1H-benzo[d]imidazol-1-yl)cyclohexyl)methanesulfonamide (**5**)*

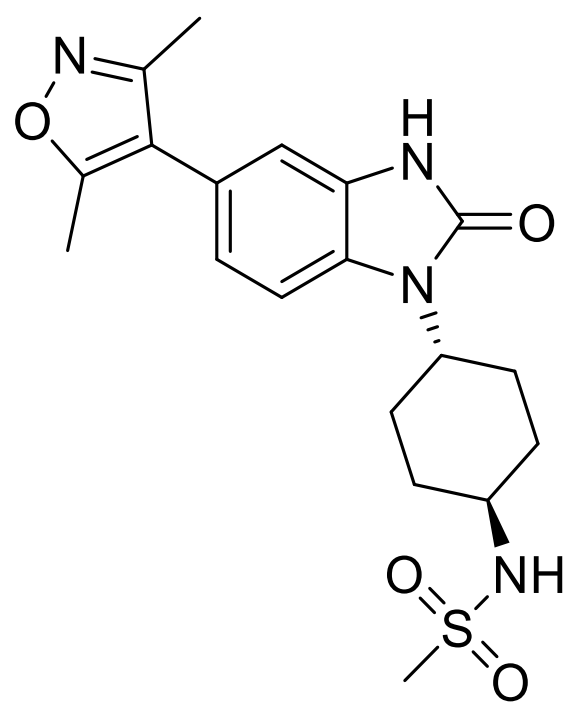

To a solution of **S6** (76.1 mg, 201 µmol) in DCM (2.2 mL) was added DMAP (4.91 mg, 40.2 µmol, 0.200 eq) and CDI (65.2 mg, 402 µmol, 2.00 eq) and the reaction was stirred at rt for 5 h. The reaction mixture was concentrated under reduced pressure. The crude material was purified by reverse-phase column chromatography (90:10-10:90, H_2O:MeCN) to yield **5** as a white solid (45.5 mg, 56%). ^1H NMR (400 MHz, DMSO-d_6) δ: 10.93 (1H, s), 7.44 (1H, d, *J* = 8.2 Hz), 7.08 (1H, d, *J* = 7.2 Hz), 6.97-6.91 (2H, m), 4.22-4.09 (1H, m), 2.95 (3H, s), 2.37 (3H, s), 2.29 (3H, q, *J* = 12.4 Hz), 2.19 (3H, s), 2.03 (2H, d, *J* = 12.4 Hz), 1.71 (2H, q, *J* = 11.6 Hz), 1.44

(2H, q, J = 10.7 Hz). $^{13}C\{^1H\}$ NMR (101 MHz, DMSO-d_6) δ: 164.6, 158.2, 153.8, 128.8, 128.6, 122.0, 121.3, 116.2, 109.4, 109.2, 50.9, 50.5, 40.8, 32.9, 27.9, 11.2, 10.5. LCMS (ESI$^+$): $C_{19}H_{24}N_4O_4S$ [M+H]$^+$ m/z calcd 405.2; found 405.1. t_R (Method 2): 4.29 min. HRMS (ESI$^+$): $C_{19}H_{24}N_4O_4S$ [M+H]$^+$ m/z calcd 405.1592; found 405.1591.

*2-(6-(3,5-Dimethylisoxazol-4-yl)-3-((1r,4r)-4-(methylsulfonamido)cyclohexyl)-2-oxo-2,3-dihydro-1H-benzo[d]imidazol-1-yl)-N-methylpropanamide (**3**)*

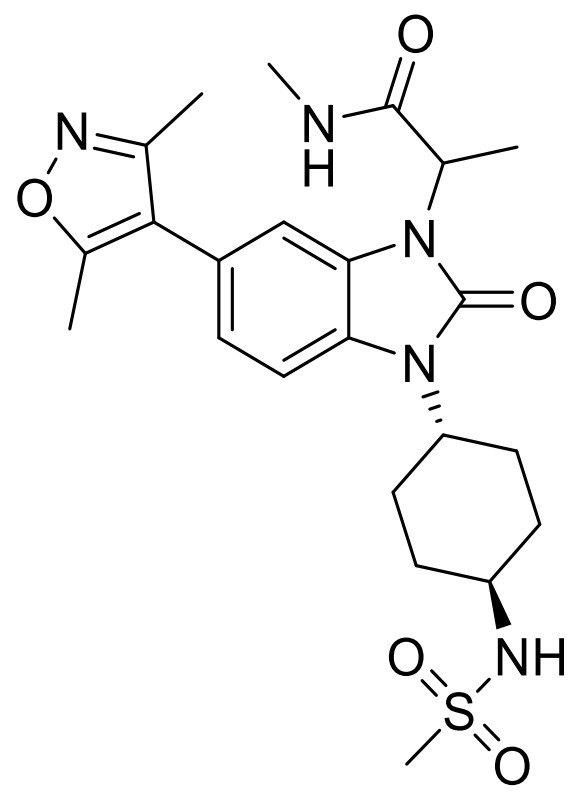

To a solution of **5** (20.0 mg, 49.4 µmol) in DMF (1.0 mL), cooled to 0 °C, was added NaH (60% pure, 2.37 mg, 59.3 µmol, 1.20 eq) and the reaction was stirred for 1 h. 2-Bromo-*N*-methylpropanamide (10.7 mg, 64.3 µmol, 1.30 eq) was added, the reaction was warmed to rt and stirred for a further 14 h. The reaction mixture was quenched with sat aq NH_4Cl (5.0 mL) and extracted with EtOAc (3 x 5.0 mL). The combined organics were washed with brine (15 mL), dried using a hydrophobic frit and concentrated under reduced pressure. The crude material was purified by reverse-phase column chromatography (90:10-10:90, H_2O:MeCN) to yield **3** as a white solid (7.90 mg, 33%). ^1H NMR (400 MHz, DMSO-d_6) δ: 8.07-7.95 (1H, m), 7.52 (1H, d, J = 8.2 Hz), 7.12-7.04 (2H, m), 7.02 (1H, d, J = 8.2 Hz), 5.03 (1H, q, J = 7.0 Hz), 4.21 (1H, t, J = 12.3 Hz), 2.95 (3H, s), 2.57 (3H, d, J = 4.6 Hz), 2.39 (3H, s), 2.35-2.24 (2H, m), 2.21 (3H, s), 2.04 (3H, d, J = 11.3 Hz), 1.76 (2H, d, J = 13.2 Hz), 1.50 (3H, d, J = 7.3 Hz), 1.48-1.38 (2H, m). $^{13}C\{^1H\}$ NMR (101 MHz, DMSO-d_6) δ: 169.4, 164.7, 158.2, 152.5, 128.4, 127.7, 121.9, 121.3, 116.1, 109.8, 109.7, 51.2, 50.9, 50.5, 40.8, 32.9, 27.9, 25.9, 15.0, 11.2, 10.4. LCMS (ESI$^+$): $C_{23}H_{31}N_5O_5S$ [M+H]$^+$ m/z calcd 490.2; found 490.2. t_R (Method 2): 4.32 min. HRMS (ESI$^+$): $C_{23}H_{31}N_5O_5S$ [M+H]$^+$ m/z calcd 490.2119; found 490.2130.

References

(1) Kelley, B. P.; Brown, S. P.; Warren, G. L.; Muchmore, S. W., POSIT: Flexible Shape-Guided Docking For Pose Prediction. *J. Chem. Inf. Model*. **2015**, *55*, 1771-1780.

(2) Petronella, K. M., Abbreviations List. In The ACS Guide to Scholarly Communication, American Chemical Society: 2019.

(3) Seal, J. T.; Atkinson, S. J.; Aylott, H.; Bamborough, P.; Chung, C.-w.; Copley, R. C. B.; Gordon, L.; Grandi, P.; Gray, J. R. J.; Harrison, L. A.; Hayhow, T. G.; Lindon, M.; Messenger, C.; Michon, A.-M.; Mitchell, D.; Preston, A.; Prinjha, R. K.; Rioja, I.; Taylor, S.; Wall, I. D.; Watson, R. J.; Woolven, J. M.; Demont, E. H. The optimization of a novel, weak bromo and extra terminal domain (BET) bromodomain fragment ligand to a potent and selective second bromodomain (BD2) inhibitor. *J. Med. Chem*. **2020**, *63*, 9093-9126. https://doi.org/10.1021/acs.jmedchem.0c00796
